# The tumor microenvironment in gastrointestinal adenocarcinomas revealed a prognostic and immunotherapeutic biomarker

**DOI:** 10.18632/aging.204463

**Published:** 2022-12-30

**Authors:** Yong Zhang, Lu Li, Feifei Chu, Lei Zhang, Li Zhang, Huili Wu, Kunkun Li

**Affiliations:** 1 Department of Gastroenterology, Zhengzhou Central Hospital Affiliated to Zhengzhou University, Zhengzhou 450007, China; 2 Medical Key Laboratory for Diagnosis and Treatment of Colorectal Cancer in Henan Province, Zhengzhou 450007, China; 3 Zhengzhou Key Laboratory for Diagnosis, Treatment and Research of Colorectal Cancer, Zhengzhou 450007, China; 4 Branch Center of Advanced Medical Research Center, Zhengzhou Central Hospital Affiliated to Zhengzhou University, Zhengzhou 450007, China

**Keywords:** tumor microenvironment, gastrointestinal adenocarcinomas, immune infiltration, prognostic, immunotherapeutic

## Abstract

Accumulated evidence has elucidated that the tumor microenvironment (TME) is great of clinical significance in predicting survival outcomes and therapeutic efficacy. Nonetheless, few studies have investigated the prognostic and immunotherapeutic signature correlated with TME phenotypes in gastrointestinal adenocarcinomas (GIAC). Here, by estimating the TME pattern of immune infiltration and expression in over 1,000 GIAC patients, we revealed three TME subgroups and identified six key differential genes. To predict the TME phenotypes, TMEscore was established and validated to be an independent prognostic factor, where the high TMEscore was characterized by immune activation and response to immunotherapy and accompanied with favorable prognosis in GIAC. Furthermore, TMEscore was confirmed to predict prognosis and immunotherapeutic response in six datasets. In summary, depicting TME landscape of GIAC patients may be beneficial for interpreting survival and immunotherapeutic response, and provide new strategies for clinical treatment of GIAC.

## INTRODUCTION

Gastrointestinal adenocarcinomas (GIAC) mainly consist of esophageal adenocarcinoma, gastric adenocarcinoma and colorectal adenocarcinoma and have the highest incidence and mortality across all kinds of malignant tumors. Approximately 1.4 million people die from GIAC each year worldwide [1]. Though GIAC has a similar epithelial tissue origin and gastrointestinal physiological environment, the clinical phenotypes and genetically molecular characteristics are quite distinct [2]. Genomic and transcriptomic analysis have further defined the heterogeneity of GIAC by identifying molecular subtypes. According to DNA mutation and copy number alteration and DNA methylation pattern, GIAC patients can categorize into five subtypes, namely, EpsteinBarr virus(EBV), chromosomal instability (CIN), microsatellite instability (MSI), hypermutated-SNV (HM-SNV) and genome stable (GS) tumors, or other four subtypes, i.e., CIMP-H, CIMP-L, EBV-CIMP and non-CIMP [3]. Recent studies suggest that nearly all GIAC tumors appear to present with four subtypes that are either characterized by canonical epithelial origin, extensive immune infiltration, metabolic dysregulation or mesenchymal gene expression signatures [4]. Although these studies have greatly enhanced the understanding of the tumor underlying mechanism, effective molecular markers to predict survival and guide treatment in specific subtypes are still lacking. Thus, it is quite essential to identify tumor subgroups, exploit prognostic and therapeutic response biomarker at the pan-digestive tract tumor level.

Calculated evidence suggests that tumor initiation and progression are not only governed by the genetic changes of cancer cells but also by tumor microenvironment (TME) factors [5]. Transformed cancer cells admixed with immune cells and stromal cellular elements form complicated TME, which significantly influences therapeutic response and clinical outcome [6]. Stromal cells (epithelial cells, and fibroblasts) as well as immune cells (macrophages, neutrophils, dendritic cells, T cells and B cells) are recurrently reported to contribute to tumor progression and metastasis when present in TME [7]. Extensive research on TME has revealed a crucial role of the immune response genes and tumor-infiltrating immune cells in patient survival outcome, tumor dissemination, relapse, metastasis, and therapeutic response to immunotherapy [8–11]. Immune checkpoint molecules PD-1, PD-L1, CTLA-4 and even their combination showed excellent predictive performance with immune checkpoint blockade response. For instance, Sun et al. investigated the relevance between the RNA expression of current biomarkers with the response of immunological therapy, and assessed predictive performance in different cancer types and therapeutic strategies [12].

Numerous studies have confirmed that immune and stromal cell infiltration and their crosstalk in the TME modulate cancer progression and are attractive therapeutic targets [13, 14]. Moreover, the effects of infiltrating immune and stromal cells on prognosis and have been extensively reported [15, 16]. Therefore, designing the therapeutics simultaneously target multiple components of the TME will benefit for increasing the likelihood of favorable patient outcomes. For predicting patient prognosis or immunotherapy response in esophageal cancer, gastric cancer and colorectal cancer, large studies have revealed many signatures or score systems based on gene expression [17–19], cell infiltration level [13, 20] or their combinations [21, 22], which providing potential biomarkers and therapeutic targets. In spite of these, no studies have been analyzed intensively in pan-gastrointestinal cancers. Meanwhile, the vast majority were not validated in additional datasets, the reliability was questionable.

Thus, we aimed to uncover a robust rating system predicting patient prognosis and immunotherapy response in GIAC based on the RNA expression of the signature genes reflecting TME. In this study, we analyzed the gene-expression profiles of GIAC patients and acquired a comprehensive landscape about TME. Based on immune infiltration and immune pathway expression patterns, we classified the GIAC into three subtypes with distinct clinical and immune characteristics. Further, we determined 6 stromal or immune genes representative for the TME of GIAC patients, and established a TME score, which could precisely predict patient survival outcome and response to immunotherapy in multiple immunotherapeutic datasets.

## MATERIALS AND METHODS

### Data source

We obtained RNASeq expression data and clinical data of 1,199 GIAC patients in PanCanAtlas [23] website from The Cancer Genome Atlas (TCGA) project. GIAC included colorectal adenocarcinoma (CRC, namely, colon adenocarcinoma (COAD), rectum adenocarcinoma (READ), gastric adenocarcinoma (GAD) and esophageal adenocarcinoma (ESAD). For validation of the prognostic value of our marker, we additionally got two datasets (GSE17536 and GSE39582) from Gene Expression Omnibus (GEO).

For validation of the immunotherapeutic predictive value of our marker, we collected six datasets, i.e., VanAll [24] (42 melanoma patients treated with CTLA4 inhibitor (CR/PR = 14, PD/SD = 23), Riaz [25] (51 melanoma patients treated with PD1 inhibitor (CR/PR = 10, PD/SD = 39), Mariathasan [26] (also named IMvigor210, 348 urothelial carcinoma patients treated with PD1 inhibitor (CR/PR = 68, PD/SD = 230), Auslander [27] (14 melanoma patients treated with PD1-CTLA4 inhibitor (CR/PR = 2, PD/SD = 12), Gide [28] (41 melanoma patients treated with PD1-CTLA4 inhibitor (CR/PR = 19, PD/SD = 22) and 32 melanoma patients treated with PD1 inhibitor(CR/PR = 21, PD/SD = 11) and Kim [29] (45 gastric cancer patients treated with PD1 inhibitor (CR/PR = 12, PD/SD = 33). The gene expression was detected using RNA transcriptional sequencing on patients prior to immunotherapy.

### Immunophenotyping for GIAC patients

We analyzed the gene-expression profiles of GIAC tumor and adjacent samples and utilized TIMER2.0 [30] and CIBERSORT [31] method to quantify infiltration level of immune cells for GIAC patients. To further investigate TME with GIAC patient classification, we firstly obtained 160 immune related signatures of 9,131 patients of multiple cancer types from panImmune [32] resource (https://gdc.cancer.gov/about-data/publications/panimmune) and only retained 1,021 GIAC (including CRC, GAD and ESAD) patients. The 160 signatures were from 7 resources, namely, Attractors (9 signatures), Bindea (25), c7atoms (32), CIBERSORT (20), ICR (3), Senbabaoglu (3) and Wolf (68) (Supplementary Tables 1, 2).

Based on the tumors with the 160 signatures, three unsupervised clustering algorithms (Lee, brunet and nsNMF) were used to identify TME patterns and classify patients. Kappa value was used to assess classification consistency of the three algorithms. Multiple classification indexes (silhouette, dispersion and cophenetic coefficient) were applied to determine the best number of clusters. These procedures were performed using the NMF R package, which adopted non-negative matrix factorization (NMF) method [33] and was repeated 1,000 times to ensure the stability of classification. Finally, the nsNMF algorithm and optimal cluster number 3 was selected, and patients were clustered into 3 TME subtypes, i.e., 403 patients from TME-C1, 276 from TME-C2, 324 from TME-C3.

Clinical and immune cell were extracted from PanCanAtlas resource (https://gdc.cancer.gov/about-data/publications/pancanatlas) (including survival information, cancer type, tumor stage, cancer cell fraction, and Shannon score) and the immune cell infiltration fractions (leukocyte, CD8 T cell, regulatory T cell, resting NK cell, and activated NK cell) estimated by CIBERSORT algorithm were obtained from panImmune resource. Fisher exact test or Pearson’s chi-square test for discrete variables and Wilcoxon rank-sum test for continuous variables were used to assess the correlation of these features and three TME subtypes.

### Differential expression analysis and functional enrichment analysis

To identify genes differentiating TME clusters, differentially expressed genes (DEGs) across these clusters were firstly identified using the R package Limma [34], which implements an empirical Bayesian approach to estimate gene-expression changes using moderated *t* tests. The 54 genes in signature A and 85 genes in signature B were used for gene enrichment analysis. Gene Ontology (GO) terms were identified with a strict cutoff of *P* < 0.01 and false discovery rate (FDR) of less than 0.05. Enriched pathways and cancer hallmarks were identified by running Gene Set Enrichment Analysis [35] (GSEA) of the adjusted expression data for all genes. Enrichment *P* values were based on 10,000 permutations and subsequently adjusted for multiple testing using the Benjamini-Hochberg (BH) procedure to control the FDR. Gene sets were downloaded from MSigDB [36] database of the Broad Institute. R function “clusterProfiler” and “enrichplot” was adopted using the clusterProfiler [37] R package was performed on TME signature A and B genes.

### Establishment of TMEscore in GIAC

For identify prognostic and immunotherapeutic gene signature, we firstly screened some representative genes distinguishing the three immune subtypes. And then the random forest classification algorithm packaged in Boruta [38] was used to perform dimension reduction in order to reduce noise or redundant genes. Univariate Cox regression analysis was utilized to determine candidate prognostic genes for PFS and OS. GIAC patients were stratified into two subgroups based on the expression of each gene above or below the median. The survival curves were plotted using Kaplan-Meier (KM) curve and the survival difference of two patient groups were estimated using log-rank test (*p* value < 0.05).

The TMEScore was established with a formula: TMEScore = exp(G_i_) + exp(G_j_), where the G_i_ and G_j_ represent the average expression of genes in signature A and signature B. TMEscore = α∑(exp_I_A_)/len(A) + β∑(exp_J_B_)/len(B), where exp_I_A_, exp_J_B_ indicates the expression of gene I in signature A, the expression of gene J in signature B; α, β were set as 1 if gene I or J were protective factors and -1 otherwise. Here, TMEscore = −(C6orf223 expression + EPHX4 expression + HES6 expression + NKD2 expression)/4 + (OLR1 expression + ONECUT2 expression)/2.

### Tissue specimens

Our in-house GIAC cohort included 96 pairs of fresh GIAC tumor and adjacent normal tissue specimens without radiotherapy or chemotherapy, which were immediately stored in liquid nitrogen after surgery (Supplementary Table 3), all enrolled subjects were pathologically diagnosed as adenocarcinoma. All specimens were collected from Zhengzhou Central Hospital Affiliated to Zhengzhou University between 2019 and 2020 and this study was approved by the Zhengzhou Central Hospital Affiliated to Zhengzhou University. All subjects have undergone rigorous screening and underwent informed consent.

### Quantitative RT-PCR (qRT-PCR)

QRT-PCR was employed to detect the RNA level of 6 genes (C6orf223, EPHX4, HES6, NKD2, OLR1 and ONECUT2). In brief, total RNAs of 96 pairs of fresh GIAC tissue specimens were extracted by Trizol method. After testing for concentration, purity, and integrity, an equal amount of RNAs was used to synthesize cDNA. Finally, SYBR Green Quantitative Kit (DBI, Germany) and 7500 Fast Quantitative PCR System (AB, USA) were used for detection. The housekeeping gene GAPDH was used as internal reference, and the relative gene expression was expressed as 2^−ΔΔCt^. Primer sequences were shown in Supplementary Table 4. The relative gene expression of the 6 genes in 96 GIAC patients (matched adjacent normal and tumor samples) were showed in Supplementary Table 5.

### Western blot

Western blot was used to detect protein levels of corresponding genes in 27 pairs of tumor and matched adjacent normal sample from our center. In brief, total protein was extracted using a lysate mixture containing RIPA and protease inhibitors. After concentration determination, an equal amount of total proteins from each sample was used for polyacrylamide gel electrophoresis. After transfer to PVDF membranes, the membranes were blocked and incubated with the primary antibody overnight at 4°C. After incubated with the secondary antibody at room temperature for 1 hour, ECL was added for exposure and development.

Primary antibodies used in this study include rabbit anti-β-tubulin (Abcam, ab6046, 1:1000 dilution for WB), rabbit anti-EPHX4 (Abcam, ab183739, 1:1000 dilution for WB), mouse anti-HES6 (Abcam, ab172800, 1:1000 dilution for WB), rabbit anti-NKD2 (CST, 2073T, 1:1000 dilution for WB), rabbit anti-OLR1 (Bioss, bs-2044R, 1:1000 dilution for WB) and anti-ONECUT2 (Bioss, bs-19643R, 1:1000 dilution for WB). A goat-anti-mouse-HRP (Bioss, bs-40296G-HRP, 1:10000 dilution for WB) antibody (Bioss, bs-19643R, 1:1000 dilution for WB) and a goat-anti-rabbit-HRP antibody (Bioss, bs-80295G-HRP, 1:10000 dilution for WB) were used in WB. The relative protein expression of the 5 genes in 27 GIAC patients (matched adjacent normal and tumor samples) were showed in Supplementary Table 6.

### Prognostic evaluation using TMEscore

For overall survival (OS), disease specified survival (DSS) and progression free survival (PFS), GIAC patients were stratified into two subgroups based on TMEscore above or below the median. We analyzed and validated the prognostic value of TMEscore in TCGA and two GEO datasets (GSE17536 and GSE39582). Receiver operating characteristic (ROC) curve analysis and Area Under Curve of ROC (AUC) was utilized to show prediction power according TMEscore and other factors. And multi-variate Cox regression analysis was employed to determine the independent prognostic factors for OS and PFS with adjustment for other potential clinicopathological factors, i.e., age, gender, and tumor stage. We adopted nomogram and calibration plot to display the predictive ability and power of multiple features using R package rms.

### Prediction of immunotherapeutic response using TMEscore

To explore the correlation between TMEscore and immunotherapeutic response, the expression profiles of six immunotherapeutic datasets were normalized into FPKM (Fragments Per Kilobase of exon model per Million mapped fragments). TMEscore was constructed using the RNA expression of the six genes. The immunotherapeutic response contained four status, complete response (CR), partial response (PR), stable disease (SD) and progression disease (PD), where the former two indicate response to immunotherapy, while the latter two indicate none-response to immunotherapy. Fisher exact test or Pearson’s chi-square test was used to measure the relevance of TMEscore (high/low) and immunotherapeutic response (responder/non-responder). Wilcoxon rank-sum test was adopted to detect the statistical difference of TMEscore between the responders and non-responders. Based on immunotherapeutic response status and patient TMEscore, ROC curve analysis and AUC were utilized to assess the immunotherapeutic predictive value of TMEscore. To predict patients' likelihood of responding to ICBs, we utilized “EaSIeR” R package [39] to estimate the immune response score based on hallmarks of immune response (CYT, TLS, IFNy, Ayers_expIS, Tcell_inflamed, Roh_IS, Davoli_IS and chemokines) in TCGA GIAC cohort and compute the integrated immune response score. Pearson correlation test was used to measure TMEscore and these immune response scores.

### Availability of data and materials

The datasets supporting our results are available in the public database GEO, TCGA and data source in method. The data of our in-house cohort is provided in Supplementary Tables.

## RESULTS

### TME landscape of GIAC

**Figure 1:**
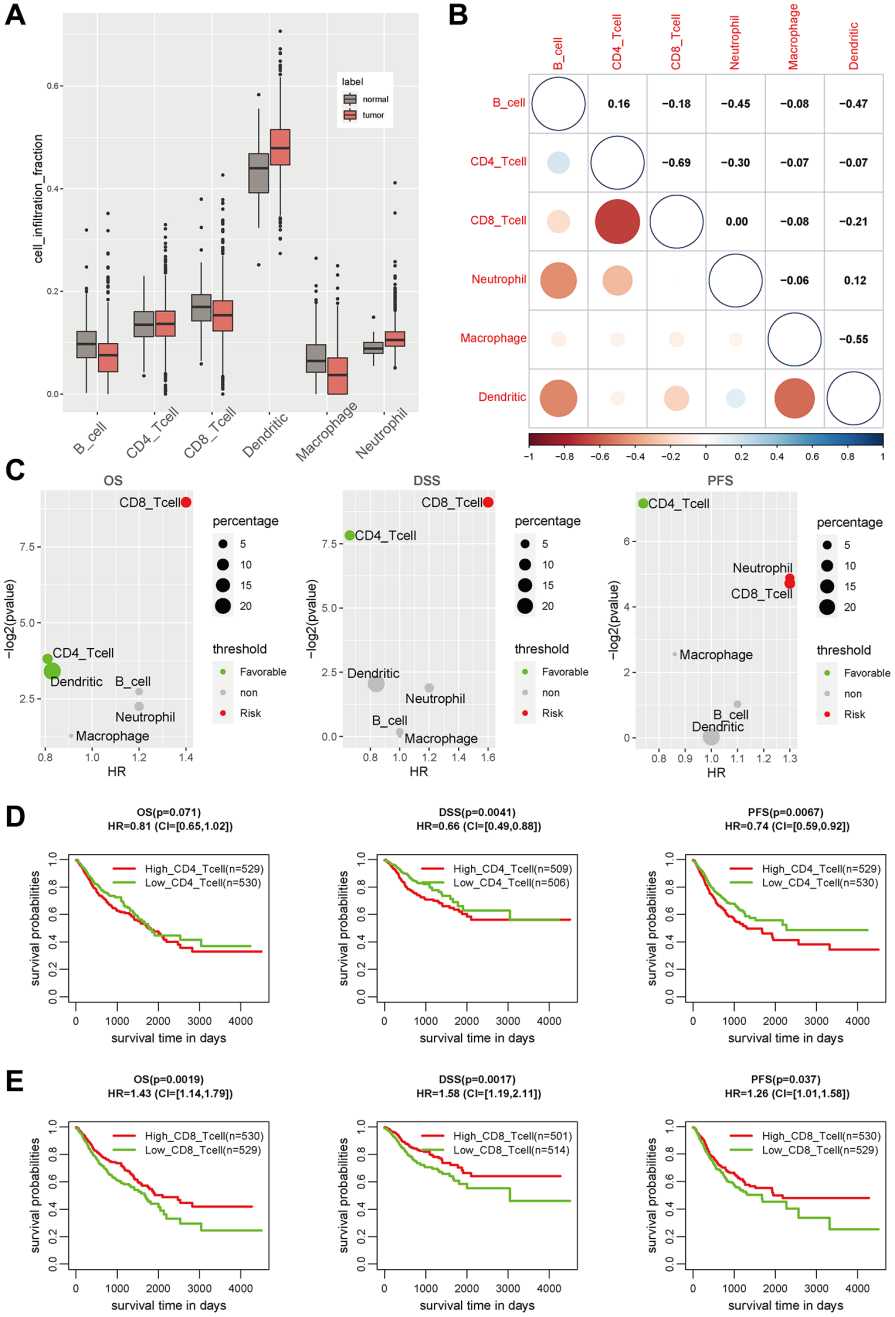
**The immune landscape in GIAC tumors.** (**A**) The cell infiltration level of six main immune cell types in GIAC tumor and adjacent normal samples. (**B**) The cell infiltration correlation of six immune cell types. (**C**) The P-value and HR of cell infiltration level of six immune cell types in survival analysis. (**D**) The KM plot of CD8 T cell infiltration in GIAC patients for OS, DSS and PFS. (**E**) The KM plot of CD4 T cell infiltration in GIAC patients for OS, DSS and PFS.

To quantify infiltration level of immune cells for GIAC patients, we analyzed the gene-expression profiles of GIAC tumor and adjacent samples by TIMER2.0. Compared with 93 adjacent normal samples, we observed that the infiltration fraction of Dendritic cell and Neutrophil in 1,068 GIAC tumors were significantly elevated, while that of B cell, CD8 T cell and macrophage were relatively decreased (Figure 1A, Supplementary Tables 7–9), suggesting the formation of a complex and differentiated tumor immune microenvironment during the progression process from normal tissue to tumor. We wondered whether there was potential for cell communication between different cell types and found that CD4 T cell and CD8 T cell (r = −0.69), Dendritic cell and B cell/macrophage (r = −0.55, −0.47), Neutrophil and B cell (r = −0.45) had significantly negative relationship (Figure 1B, Supplementary Table 10). For OS, DSS and PFS, GIAC patients with high infiltration of CD8 T cell had favor survival rate, while those with high infiltration of CD4 T cell had poor survival rate (Figure 1C–1E, Supplementary Table 11). The TME landscape depicted the infiltration fraction and interactions of GIAC immune cells, as well as their effects on patient survival outcome.

### Three GIAC subtypes were determined based on TME pattern

**Figure 2:**
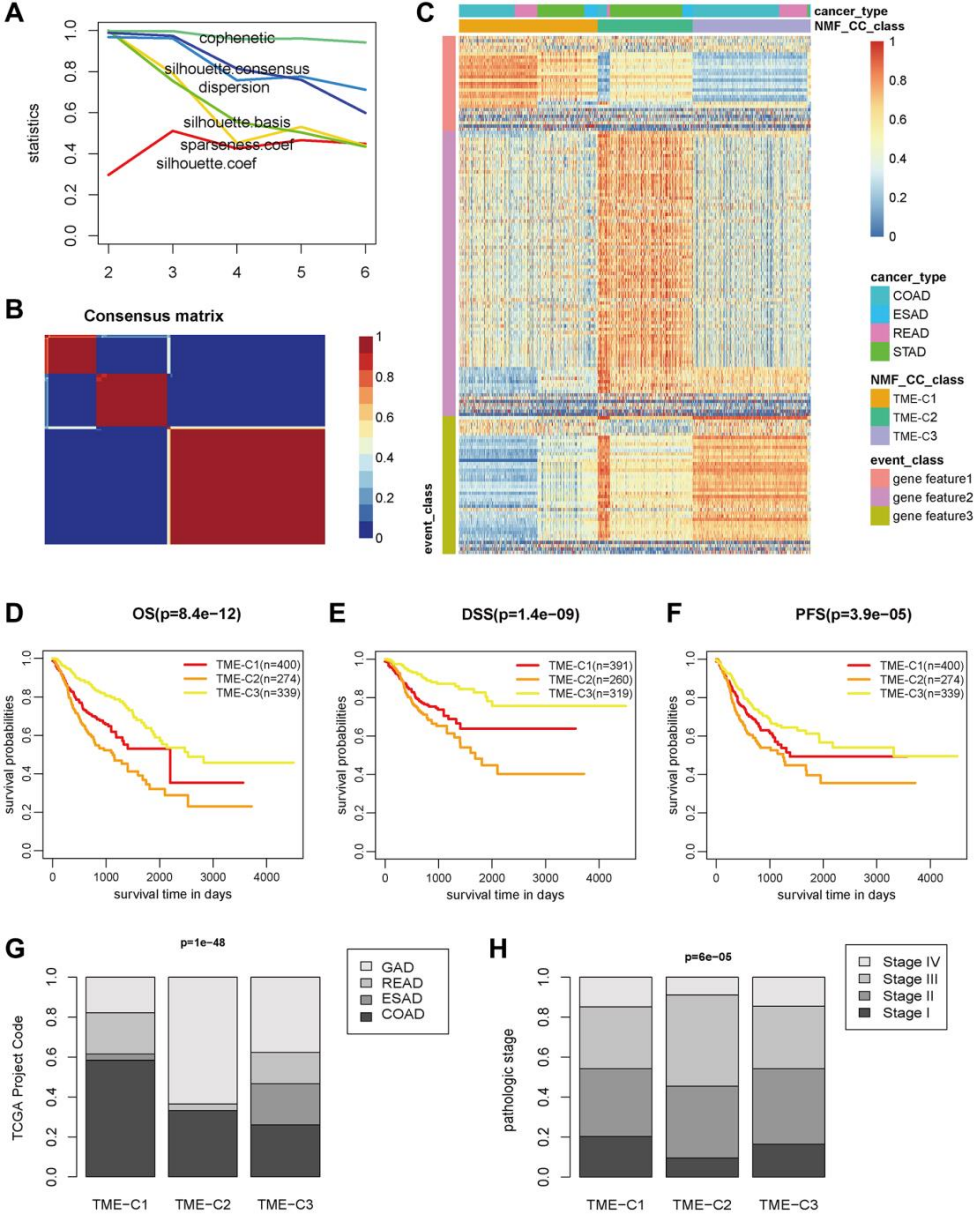
**The three TME subtypes in GIAC tumors.** (**A**) The classification indexes of different clusters in NMF results. (**B**) The consensus matrix when the cluster set as 3. (**C**) The heatmap of immune features for three TME subtypes determined by NMF classification analysis. (**D**–**F**) The KM plot of three TME subtypes in GIAC patients for OS, DSS and PFS. (**G**, **H**) The proportion of cancer types and tumor grade for the three TME subtypes.

Immunotyping can mirror the immune status in tumors and their TME, and thus benefit for identifying suitable patients for immunotherapy. Based on the TME pattern of 1,100 GIAC patients with matched 160 immune features from panImmune project, unsupervised clustering was performed using three distinct algorithms (Lee, brunet and nsNMF), and their classification consistency (Kappa value) was close to and above 0.7 (Supplementary Table 12), which indicated the stability of classification based on TME pattern of immune infiltration and expression. The nsNMF algorithm was selected due to the highest average classification consistency than the others, and the optimal cluster number was set to three according to multiple clustering indicators (Figure 2A, 2B, Supplementary Figures 1, 2, Supplementary Table 13). The GIAC patients from three main groups (termed as TME C1 (403 patients), C2 (276 patients) and C3 (324 patients) were determined (Figure 2C). They showed significant differences in OS, DSS and PFS, where TME C3 and C2 had the best and worst survival, respectively (Figure 2D–2F). When considering the cancer type, COAD and READ were almost evenly distributed in the three groups, while TME C2 and C3 were enriched in GAD and ESAD, respectively (Figure 2G, Supplementary Table 14). Meanwhile, TME C2 had higher fraction of patients with tumor stage III and IV (Figure 2H, Supplementary Table 14), which was consistent with the above survival outcome, reflecting its high degree of malignancy.

### TME-C2 subtype was associated with tumor immunity

**Figure 3:**
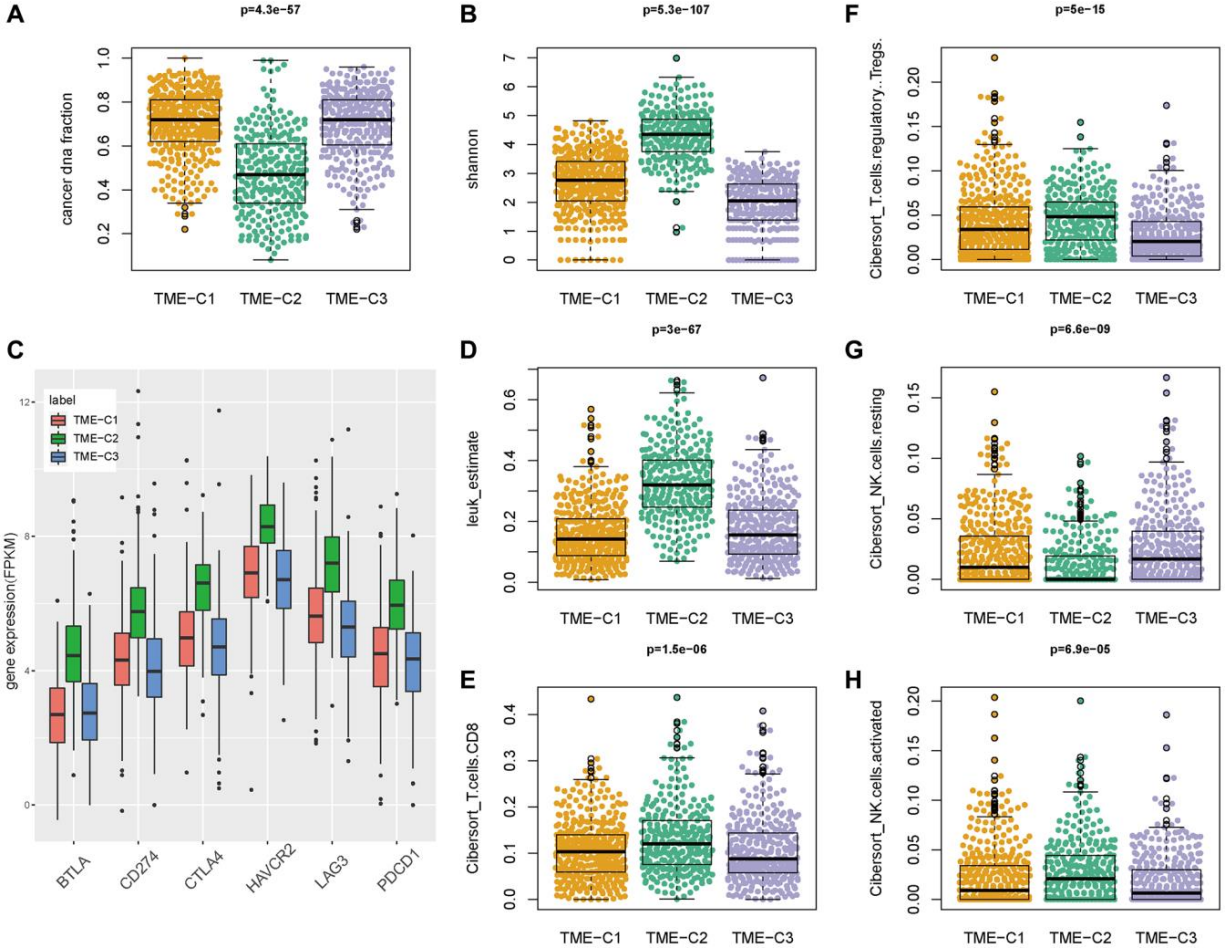
**TME-C2 subtype was associated with tumor immunity.** (**A**, **B**) The cancer DNA fraction and Shannon score across three TME subtypes. (**C**) The expression of current approved and potential immunotherapy targets (PDCD1, CD274, CTLA4, HAVCR2, LAG3 and BTLA) across three TME subtypes. (**D**–**H**) The immune cell infiltration fraction (leukocyte, CD8 T cell, regulatory T cell, resting NK cell, and activated NK cell) across three TME subtypes.

By association with genomic characteristics, we discovered that TME C2 was closely associated with lower cancer DNA fraction and larger Shannon score (Figure 3A, 3B, Supplementary Table 14), suggesting relatively its high tumor heterogeneity. Considering the crucial role of immune checkpoints (ICPs) and immunogenic cell death (ICD) modulators in cancer immunity, we next investigated their expression level in the three subtypes. Most of the ICPs and ICDs related genes were differentially expressed between the immune subtypes (Supplementary Figures 3, 4). For instance, the current approved and potential immunotherapy targets, PDCD1 (also known as PD-1), CD274 (also known as PD-L1), CTLA4, HAVCR2 (also known as TIM3), LAG3 and BTLA’s expression were significantly elevated in TME-C2 (Figure 3C, Supplementary Tables 15, 16). In the context of cancer, PD-L1 is usually highly expressed on tumor cells, thereby evading immune surveillance, but it has also been reported that high PD-L1 expression can make tumor cells more sensitive to PD-1/PD-L1 inhibitors. TME C2 was also related to the higher fraction of leukocyte, CD8 T cell, regulatory T cell and activated NK cell, and the lower fraction of resting NK cell (Figure 3D–3H, Supplementary Table 17), indicating their potential benefit for immunotherapy, such as PD1 inhibitors or other combination therapies. Therefore, immunotyping could reflect the level of immune modulators and guild the selection of population suitable for immunotherapy.

### Two TME signatures were involved in stromal and immune functions

**Figure 4:**
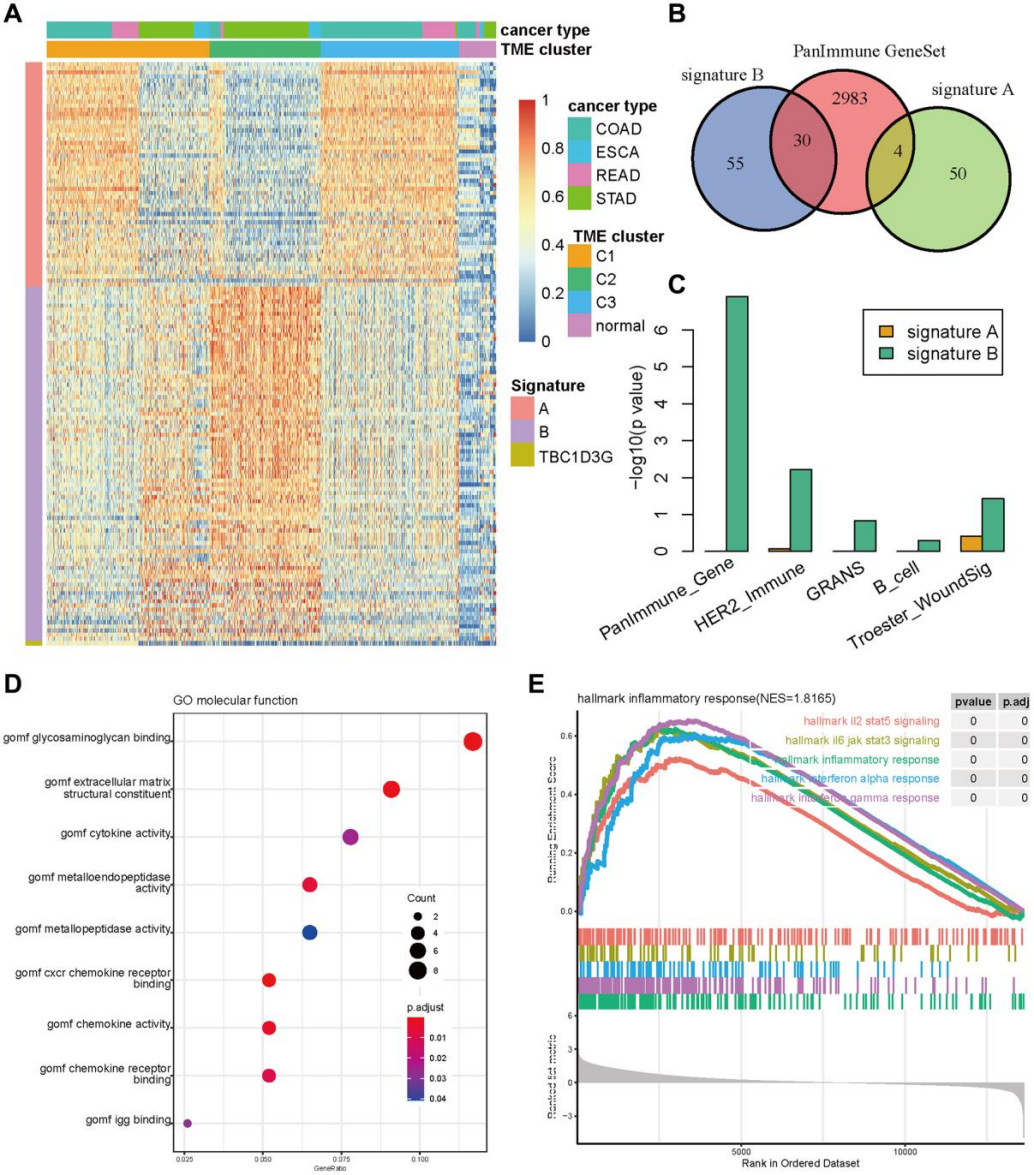
**The signature genes differentiating TME subtypes.** (**A**) Gene expression of signature A, B and TBC1D3G in three TME subtypes and normal samples. (**B**) The Venn diagram of signature A, B and PanImmune gene sets. (**C**) The enrichment of signature A and B in immune related gene sets. (**D**) The bubble diagram of signature A enriched in GO terms. (**E**) The GSEA plot of signature A enriched in cancer hallmark gene sets.

To uncover the underlying biological mechanism of the TME phenotypes, high-confidence differential expressed genes (DEGs) were acquired across TME phenotypes, where TBC1D3G was specifically highly expressed in TME C1, 54 genes (termed as signature A) was expressed in TME C2, and 85 genes (termed as signature B) was expressed in TME C3 (Figure 4A, Supplementary Table 18). TBC1D3G was reported to regulate the payload of macrophage-released extracellular vesicles to mediate inflammation [40]. We focused our attention on signature A and B, which showed enrichment in distinct molecular functions by literature annotation and overlapping with panImmune gene sets (Figure 4B, Supplementary Table 19). Signature B was involved in tumor immunity in GIAC, while signature A was involved in stromal-related function (Figure 4C, Supplementary Tables 20, 21). GO functional enrichment analysis also proved that signature B, instead of signature A, was enriched in chemokine activity and chemokine receptor binding (Figure 4D). Chemokines and their receptors play a key role in tumor growth, invasion and metastasis, as well as differentiation and development of immune cells and the regulation of immune response [41]. Gene-set enrichment analysis (GSEA) also demonstrated that signature B was indeed involved in inflammatory and immune processes (Figure 4E).

### TMEscore was a prognostic biomarker for predicting GIAC patient outcome

**Figure 5:**
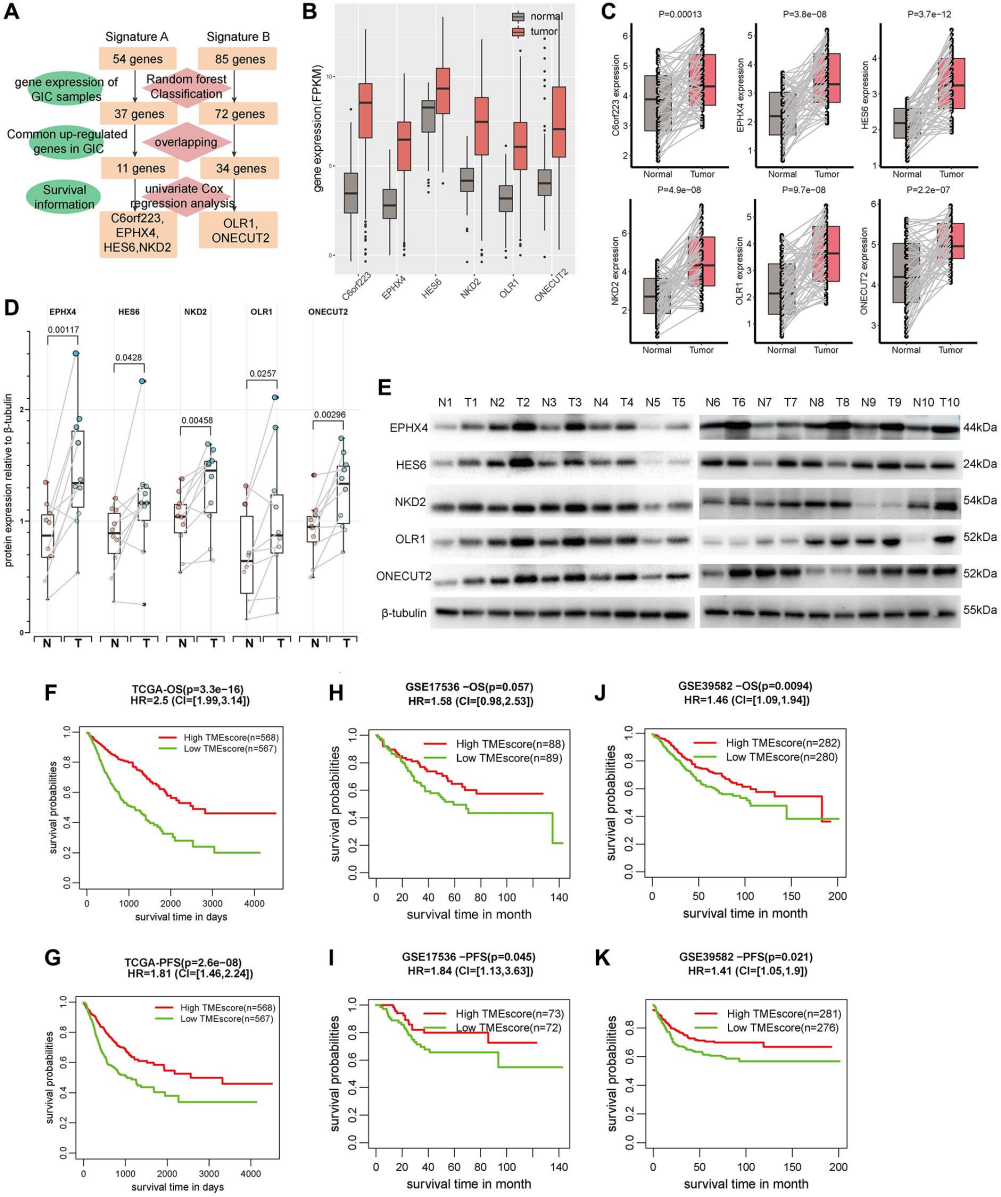
**TMEscore was an independent factor for predicting patient survival**. (**A**) The identification flowchart of six prognostic genes (C6orf223, EPHX4, HES6, NKD2 OLR1 and ONECUT2). (**B**) The expression of the six genes in GIAC tumor and normal samples from TCGA cohort. (**C**) The RNA expression of the six genes in 55 pairs of CRC tumor and normal samples from our center (GAD and ESAD see in Supplementary Figures 7, 8). (**D**) The expression of the five proteins in 10 pairs of CRC tumor and normal samples from our center (GAD and ESAD see in Supplementary Figures 9, 10). (**E**) The WB image of protein expression for CRC (GAD and ESAD see in Supplementary Figures 11, 12). (**F**–**K**) The survival time (OS and PFS) of patients with high/low TMEscore in TCGA cohort (**F**, **G**), GSE17536 (**H**, **I**) and GSE39582 (**J**, **K**).

To refine the gene signature A and B, the random forest classification algorithm was used to perform dimension reduction in order to reduce noise or redundant genes. Overlapping with the common up-regulated genes in three cancer types (CRC, GAD and ESAD), 11 genes in signature A and 34 genes in signature B were kept. To investigate the relationship between these genes with patient survival, univariate Cox regression analysis was performed and 6 prognostic genes (C6orf223, EPHX4, HES6 and NKD2 in signature A, OLR1 and ONECUT2 in signature B) were identified to correlate to both patient OS and PFS (Figure 5A, Supplementary Table 22). The patients with high expression of C6orf223, EPHX4, HES6 and NKD2 had significantly longer survival time than other patients, while patients with high expression of OLR1 and ONECUT2 had significantly shorter survival time (Supplementary Figures 5, 6), which suggested C6orf223, EPHX4, HES6 and NKD2 were protective factors and OLR1 and ONECUT2 were risk factors.

We then focused on their expression status at the RNA and protein levels. Their RNA expression in GIAC tumors were significantly elevated compared with adjacent normal samples in TCGA cohort (Figure 5B). We subsequently explored their RNA expression status in 96 GIAC patients (55 CRC, 32 GAD and 9 ESAD) from our in-house cohort by qRT-PCR assay. Their RNA expression levels were significantly up-regulated in GIAC compared with matched adjacent normal samples (Figure 5C, Supplementary Figures 7, 8). Since C6orf223 was annotated as a long noncoding RNA in GeneCards, thus we investigated the protein expression status of other five protein-coding genes in 27 GIAC patients (10 CRC, 10 GAD and 7 ESAD) from our in-house cohort by Western blot assay. Their protein expression levels were also significantly elevated in GIAC compared with matched adjacent normal samples (Figure 5D, Supplementary Figures 9, 10; Figure 5E and Supplementary Figures 11, 12). Similar results in GIAC tumors were also confirmed in multiple previous studies (Supplementary Table 23). Interestingly, though EPHX4 has hardly been studied in GIAC, the immunohistochemical (IHC) data from the HPA database revealed that its staining showed moderate to strong cytoplasmic immunoreactivity in CRC and GAD, and weak immunoreactivity in normal colon/rectum and stomach tissues (Supplementary Figures 13, 14).

We used the weighted average expression of these six genes to construct a TMEscore. Univariate and multi-variate Cox regression analysis showed TMEscore was as an independent prognostic factor in GIAC even considering the confounding factors such as age, gender and tumor stage (Supplementary Tables 24, 25). In TCGA, we observed that the patients with high TMEscore had significantly better prognosis than others, suggesting the predictive value of TMEscore for patient survival in GIAC (HR = 2.5, *P* = 3.3e-16 for OS and HR = 1.81, *P* = 2.6e-8 for PFS, Figure 5F, 5G). The nomogram analysis for OS also showed the good predictive ability of TMEscore as well as gender and tumor stage (Supplementary Figure 15), thus ROC curve analysis based on the independent prognostic factors (TMEscore, age, gender, and tumor stage) was performed. The AUC of TMEscore (0.81, 0.8, and 0.8 for 1-, 3- and 5-year OS) were obviously higher that of age, gender, and tumor stage (Supplementary Figure 16). The calibration plot also showed good consistency between observation and predictive values for 1-, 3- and 5-year OS (Supplementary Figure 17). The nomogram analysis and ROC curve analysis for PFS had similar results (Supplementary Figures 18–20). In addition, we analyzed the relevance of TMEscore and survival outcome in GSE17536 and GSE39582 from GEO and validated the prognostic value of TMEscore (Figure 5H–5K). These results declared that TMEscore was a potential biomarker for predicting GIAC patient survival.

### TMEscore predicted patient survival and immunotherapeutic benefits

In order to illustrate the role of TMEscore in tumor immunotherapy, we collected six immunotherapy datasets (Gide, Mariathasan, Auslander, Riaz, VanAll and Kim cohort) in melanoma, urothelial carcinoma and gastric cancer. Then we computed TMEscore based on the RNA expression of the six genes (C6orf223, EPHX4, HES6, NKD2, OLR1 and ONECUT2) and assessed TMEscore’s relevance of patient survival time and immunotherapy response.

**Figure 6:**
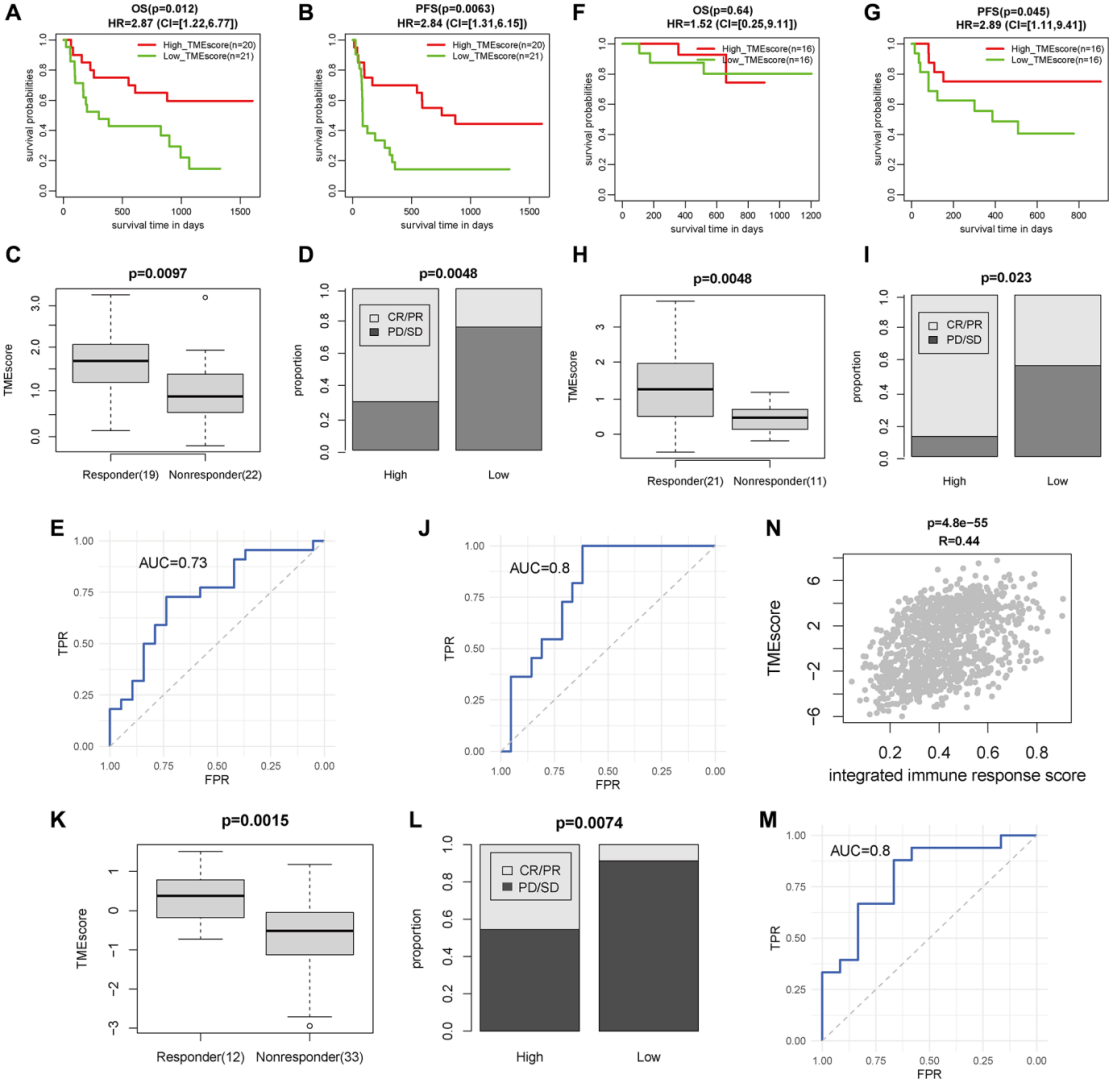
**TMEscore was a prognostic and immunotherapeutic biomarker in Gide, Kim and TCGA GIAC cohort.** (**A**–**C**) The survival time (OS and PFS) and response rate of patients with high/low TMEscore for 41 patients treated with PD1+CTLA4 inhibitor in Gide cohort. (**D**) The TMEscore of patients with different response status for patients treated with PD1+CTLA4 inhibitor. (**E**) The ROC curve of TMEscore predicting immunotherapeutic response for patients treated with PD1+CTLA4 inhibitor. (**F**–**H**) The survival time (OS and PFS) and response rate of patients with high/low TMEscore for 32 patients treated with PD1 inhibitor in Gide cohort. (**I**) The TMEscore of patients with different response status for patients treated with PD1 inhibitor. (**J**) The ROC curve of TMEscore predicting immunotherapeutic response for patients treated with PD1 inhibitor. (**K**) The response rate of patients with high/low TMEscore for 45 gastric cancer patients treated with PD1 inhibitor in Kim cohort. (**L**) The TMEscore of patients with different response status for patients treated with PD1 inhibitor. (**M**) The ROC curve of TMEscore predicting immunotherapeutic response for patients treated with PD1 inhibitor. (**N**) The correlation plot of TMEscore with integrated immune response score in TCGA GIAC cohort.

In Gide cohort, the 41 melanoma patients treated with PD1-CTLA4 combination inhibitor with high TMEscore had significantly better OS and PFS than other patients (*P* = 0.0012, HR = 2.387, CI = [1.22, 6.77] for OS; *P* = 0.0063, HR = 1.31, CI = [1.31, 6.15] for PFS, respectively, Figure 6A, 6B). TMEscore of the responders (*n* = 19) was apparently higher than that of non-responders (*n* = 22) (*P* = 0.0097, Figure 6C), and the patients with high TMEscore had significantly higher response rate than those with low TMEscore (*P* = 0.0048, Figure 6D). The TMEscore had a good immunotherapeutic predictive value (AUC = 0.73, Figure 6E). Similarly, the 32 melanoma patients treated with PD1 inhibitor with high TMEscore had significantly better PFS than other patients (*P* = 0.045, HR = 2.89, CI = [1.11, 9.41], Figure 6G), while OS had no statistical difference (Figure 6F). TMEscore of the responders (*n* = 21) was higher than that of non-responders (*n* = 11) (*P* = 0.0048, Figure 6H), and the patients with high TMEscore had increased response rate compared with the others (*P* = 0.0023, Figure 6I). TMEscore also had an outstanding immunotherapeutic predictive value (AUC = 0.8, Figure 6J). We observed a similar phenomenon in the other four data sets (Mariathasan, Auslander, Riaz, and VanAll cohort) (Supplementary Figure 21).

Besides, in Kim cohort, 45 gastric cancer patients were treated with PD1 inhibitor and 12 patients have complete response or partial response. The TMEscore of the responders (*n* = 33) was higher than that of non-responders (*n* = 12) (*P* = 0.0015, Figure 6K), and the patients with high TMEscore had increased response rate compared with the others (*P* = 0.0074, Figure 6L). The immunotherapeutic predictive value was AUC = 0.8 (Figure 6M). Furthermore, TMEscore was highly correlated with eight response scores based on hallmarks of immune response estimated by “EaSIeR” and the integrated score in TCGA GIAC dataset (Supplementary Figure 22, Figure 6N). In summary, these results demonstrated that TMEscore could predict tumor survival and immunotherapy response status.

## DISCUSSION

Despite numerous clinical trials about gastrointestinal cancer treatment in the last two decades, especially for metastatic patients, the clinical outcome is still not optimistic, and the survival usually is less than 30 months [42]. Innovative ideas, such as the gut microbiota imbalance and tumor immune microenvironment, have been introduced into the basic research of GIAC and tentative clinical treatment [43, 44]. In addition to traditional radiotherapy and chemotherapy, immunotherapy, cellular therapy, molecular targeted therapy and microbial therapy have developed rapidly [45], thus forming some experience and consensus guiding clinical practice. Hence, the development of novel and effective strategies to control GIAC is an urgent need in GIAC prognosis and treatment.

Immune checkpoint blocking (ICB) therapy has caused a great change of the therapeutic landscape, making some advanced-stage cancer patients achieve clinical benefits [46]. It is quite attracting and necessary to develop biomarkers for ICB response for clinical trials and applications [47]. Sun et al. systematically assessed the predictive power of 22 current transcriptomic biomarkers for ICB responses involving immune checkpoints and lymphocyte infiltration in multiple ICB treatment baseline datasets [12]. They revealed that these biomarkers exhibited distinct predictive value for ICB response, where some performed superior overwhelmingly or slightly just in certain circumstances. Unfortunately, some accepted biomarkers still did not have any predictive value of ICB response in benchmark datasets, indicating the possibility of the combination of multiple biomarkers for predicting ICB response in future.

The underlying mechanisms of immunotherapy have been widespread explored and some consensus has emerged. When T cell is continuously stimulated, T cell will become exhausted and continuously express high PD-1. In the tumor microenvironment, tumor cells can express PD-L1 or PD-L2. As a result, T cell function is continuously inhibited, which makes it unable to kill tumor cells [48]. One-way cancer cells evade destruction by the immune system is through ligands attached to the PD-1 protein of T cells. When the ligand binds to PD-1, T cells are unable to detect tumors and deliver signal the immune system to attack them [49]. The mechanism of PD-L1/PD-1 antibody is that tumor cells use PD-L1 to bind to the PD-1 of T cells, “trick” T cells, evade the recognition of T cells, and continue to roam in the body. However, PD-L1/PD-1 inhibitors can help T cells uncover the hypocrisy of tumor cells and restore their recognition and killing of tumor cells [50]. In recent years, immunotherapy has shown great success in improving response to some solid cancers, especially melanoma [51]. Thus, numerous clinical and preclinical studies have motivated researchers to focus their attention on gastrointestinal cancers, expecting that patients will benefit from immunotherapy, as represented by PD1 and CTLA4 blockers. In 2021, PD-1 antibody combined with chemotherapy achieved a substantial breakthrough in the first-line treatment of esophageal cancer. In Keynote-590 phase 3 trial, first-line PD-L1 inhibitor pembrolizumab plus 5-fluorouracil or cisplatin significantly improved median OS and PFS in esophageal cancer patients [52]. The first-line treatment regimen of PD-1 monoclonal antibody combined with chemotherapy or HER2 monoclonal antibody were found to improve the survival and effective rate of HER2-negative gastric cancer patients. In CheckMate 649 phase 3 trial, PD-L1 inhibitor nivolumab combined with FOLFOX or CAPE-OX as first-line therapy had better OS and PFS than first-line chemotherapy [53]. For MSI-H/dMMR CRC, immune monotherapy showed consistent advantages and moved forward to become the new standard of first-line treatment. In Keynote-177 phase 3 trial, pembrolizumab as first-line therapy in MSI-H/dMMR metastatic CRC tended to reduce the risk of death and increase PFS compared with standard therapy [54]. The success of these studies heralded a shift in the treatment strategy for certain types of gastrointestinal cancer, and prompting the National Comprehensive Cancer Network (NCCN) or Chinese Society of Clinical Oncology (CSCO) guidelines to incorporate new protocols.

In CRC, the level of T cell infiltration to tumor is directly related to the therapeutic effect of tumor patients [55], suggesting that immune cell infiltration in tumor microenvironment plays a key role in inhibiting tumor growth. The MSI-H/dMMR CRC showed a higher accumulation of tumor mutations, accompanied by a higher level of immune cell infiltration. Pembrolizumab, nivolumab, and ipilimumab were approved to treat MSI-H/dMMR tumors mainly due to their severe infiltration with CD8+/CD4+ T cells T cells [56]. Metastatic CRC generally has a higher level of mutation accumulation and thus has a response effect to immunotherapy. Although advanced (Stage IV) MSI-H/dMMR tumors account for 2–4% of all metastatic CRC, they have high expression of PD1, PDL1 and CTLA4, making this subtype more sensitive to immune checkpoint inhibitors. The low mutation load and immune cell infiltration are considered to be the main causes of immune resistance and non-response to immunotherapy for MSI-L/pMMR CRC [57]. Currently, immune checkpoint inhibitors in combination with other therapies are being explored in various preclinical and clinical trials for treatment of CRC with MSI-L/pMMR [58].

Although we have revealed TMEscore to be a biomarker for predicting prognosis and immunotherapeutic response, there are still some limitations and deficiencies in this study. First, the expression and biological function of the six key genes need further experimental investigation, including expression assays in more patients, as well as cell and animal function assays. Second, at present, immunotherapy datasets mainly focus on melanoma and urothelial carcinoma, there is indeed few immunotherapy datasets in GIAC patients except for Kim cohort (gastric cancer). We consider that many antitumor drugs have a broad spectrum and sometimes can be applied in multiple tumors, which is no exception for the immune-checkpoint inhibitors targeting PD1 and CTLA4. Thus, we extra used the transcriptional sequencing data of GIAC patients and immunotherapy response status of melanoma and urothelial carcinoma patients to assess the value of TMEscore. Though this method often used in previous studies [59–64] can be serve as a tentative and preliminary expansion application, it is indeed biased and not rigorous. When relevant data is publicly available in the future, we can precisely assess the predicted value of TMEscore in matched datasets. Finally, whether TMEscore can be serve as a prognostic and immunotherapeutic biomarker of pan-cancer is worth exploring in multiple human cancers.

In conclusion, we comprehensively analyzed the pattern of immune infiltration and immune pathway expression in over 1,000 GIAC patients and revealed three TME associated patient subgroups accompanied with quite distinct immune and clinicopathologic features. Based on the RNA expression of six key genes from two immune/stromal signatures, TMEscore was established and validated to be predictive to patient survival outcome and response to immune-checkpoint inhibitors in multiple immunotherapeutic datasets. In summary, depicting a comprehensive TME landscape will benefit for understanding the underlying mechanisms in GIAC, such as cell communication and immunosuppression or activation. TMEscore will be useful to account for the responses of GIAC to immunotherapies and provide new strategies for the treatment of cancers.

## Supplementary Materials

Supplementary Figures

Supplementary Tables 1-3, 5-8, 12-15, 17, 18, 20, 21

Supplementary Tables 4, 9-11, 16, 19, 22-25
