# Supplementary material for: The tumor microenvironment in gastrointestinal adenocarcinomas revealed a prognostic and immunotherapeutic biomarker
Source: Aging (Albany NY). 2022 Dec 30;14(24):10171–216. doi: 10.18632/aging.204463 (PMC9831739; doi:10.18632/aging.204463)
Supplement: Supplementary Figures [file aging-14-204463-s001.pdf]

SUPPLEMENTARY FIGURES

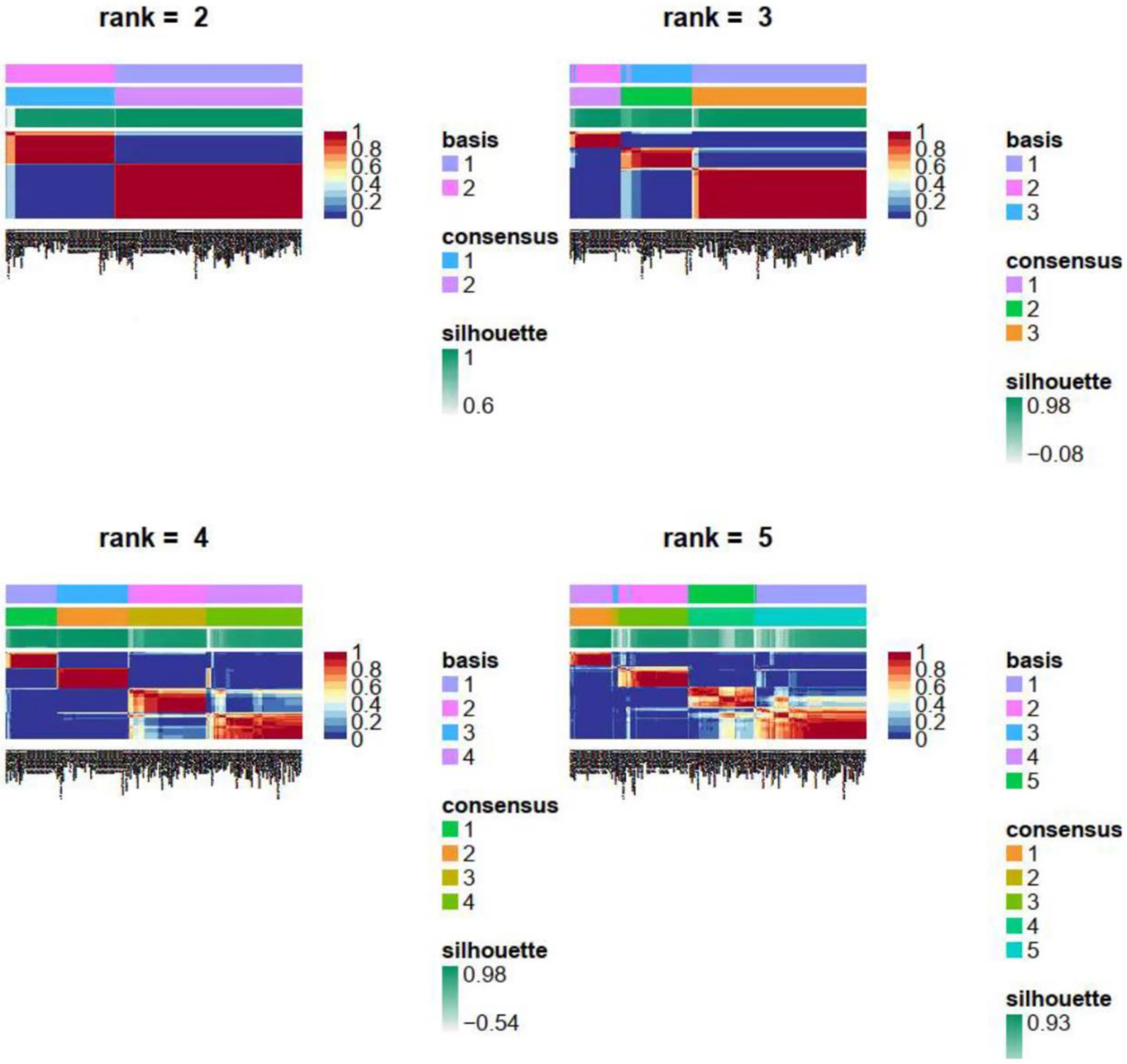

Supplementary Figure 1. The consensus map of nsNMF clustering for cluster number = 2,3,4,5.

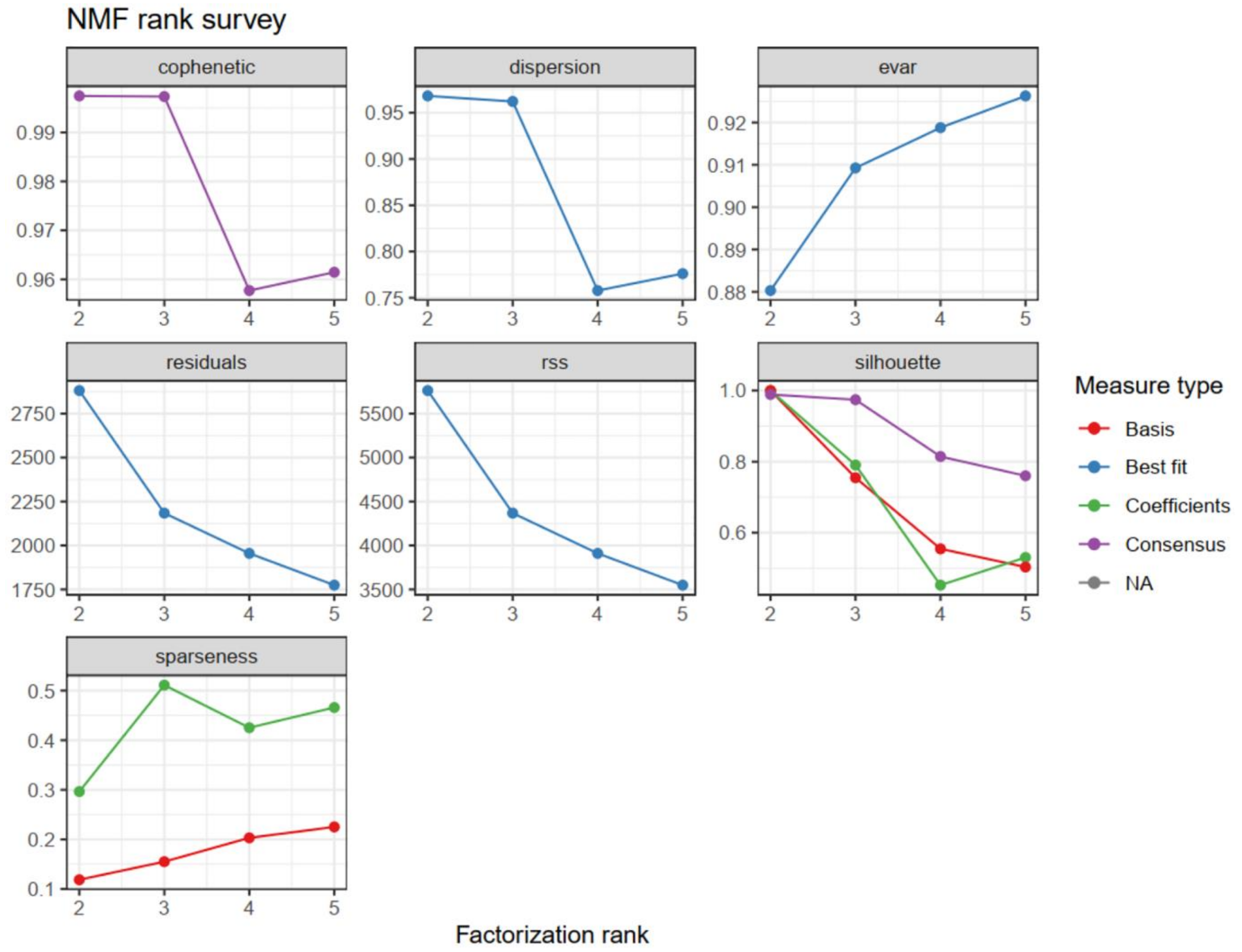

Supplementary Figure 2. The cluster indicators of nsNMF clustering for cluster number = 2,3,4,5.

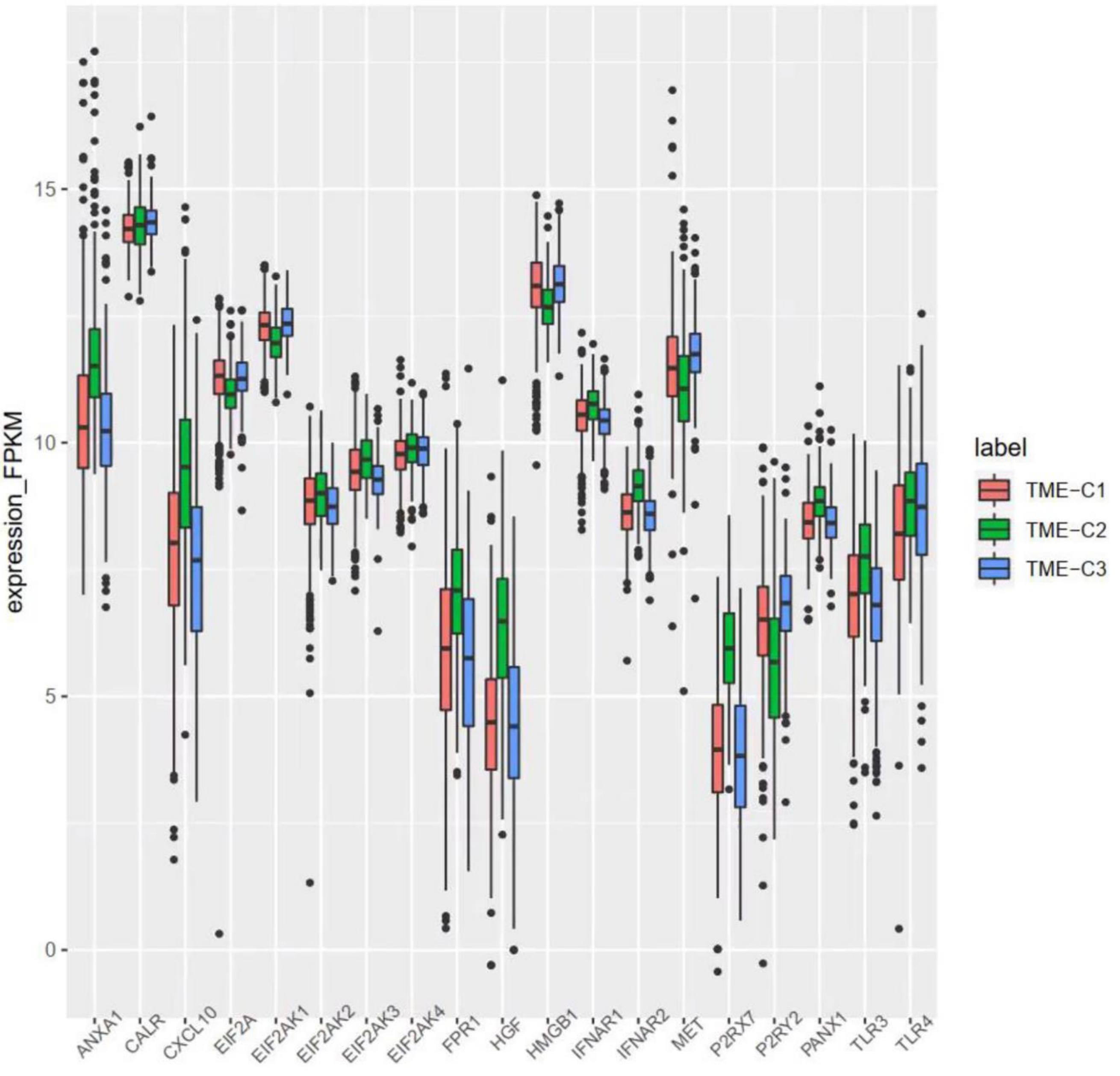

Supplementary Figure 3. The expression of 21 immunogenic cell death (ICD) modulators across three immune subtypes.

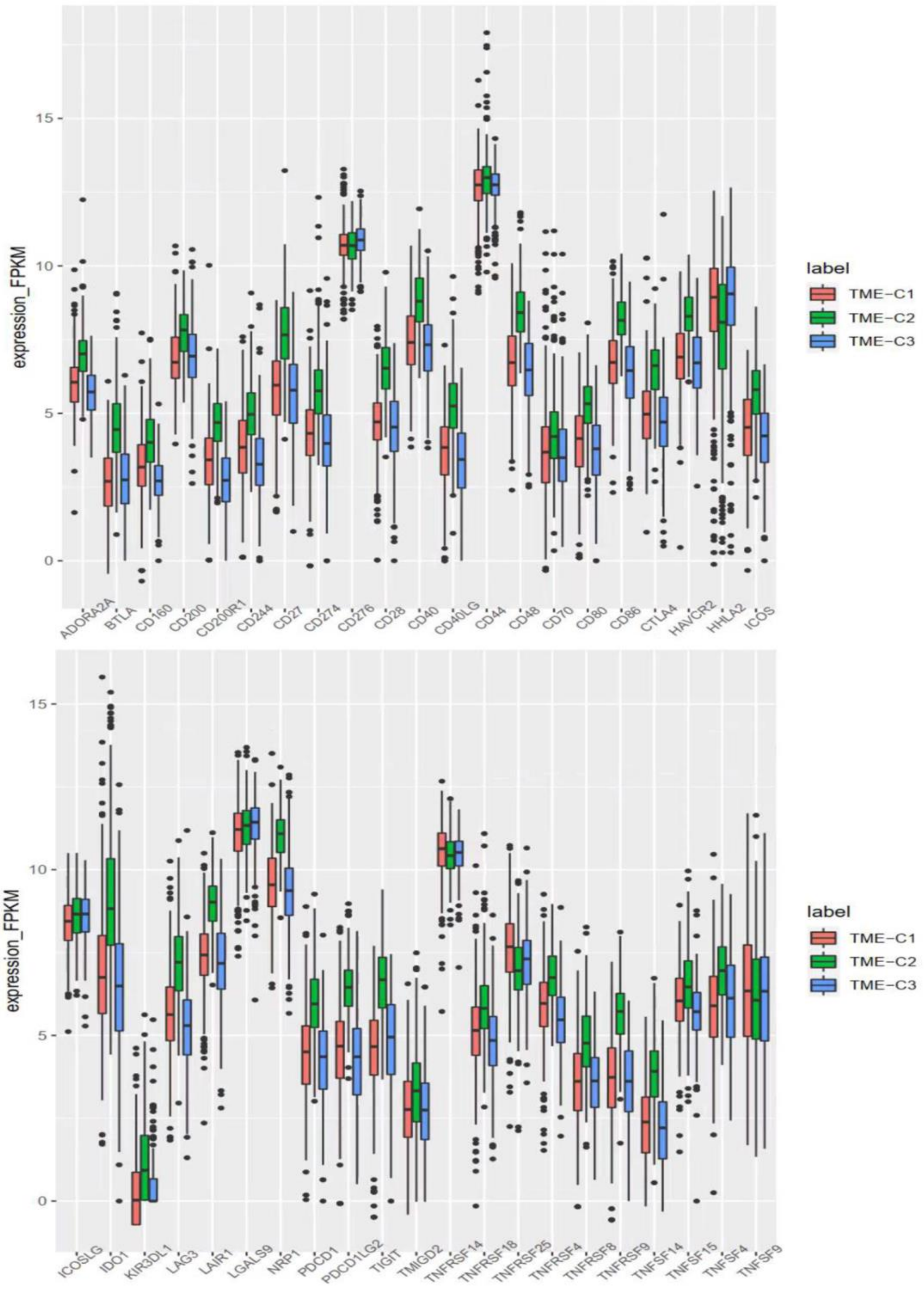

Supplementary Figure 4. The expression of 40 immune checkpoints (ICPs) across three immune subtypes.

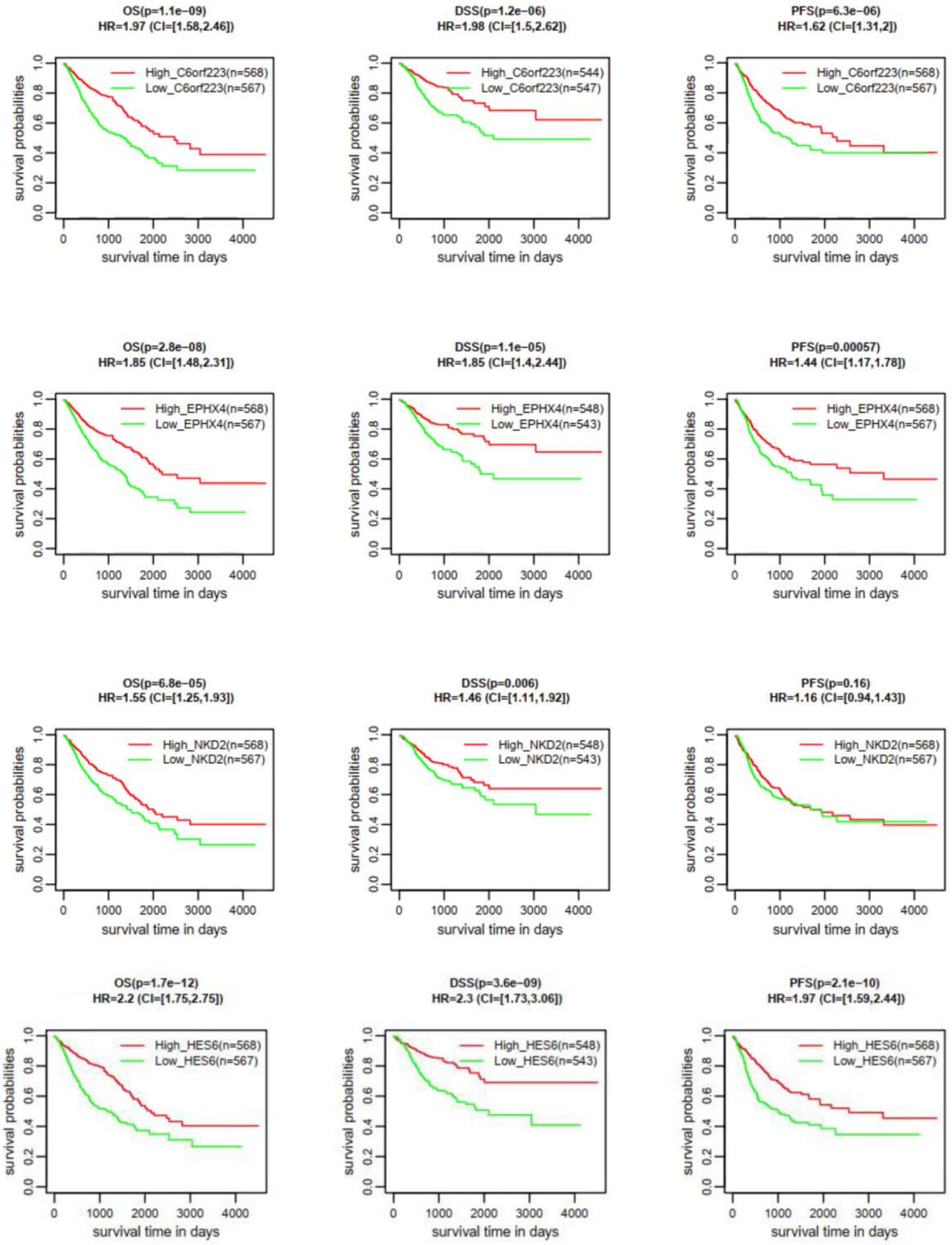

Supplementary Figure 5. The survival plot of patient groups with different C6orf223, EPHX4, HES6 and NKD2 expression.

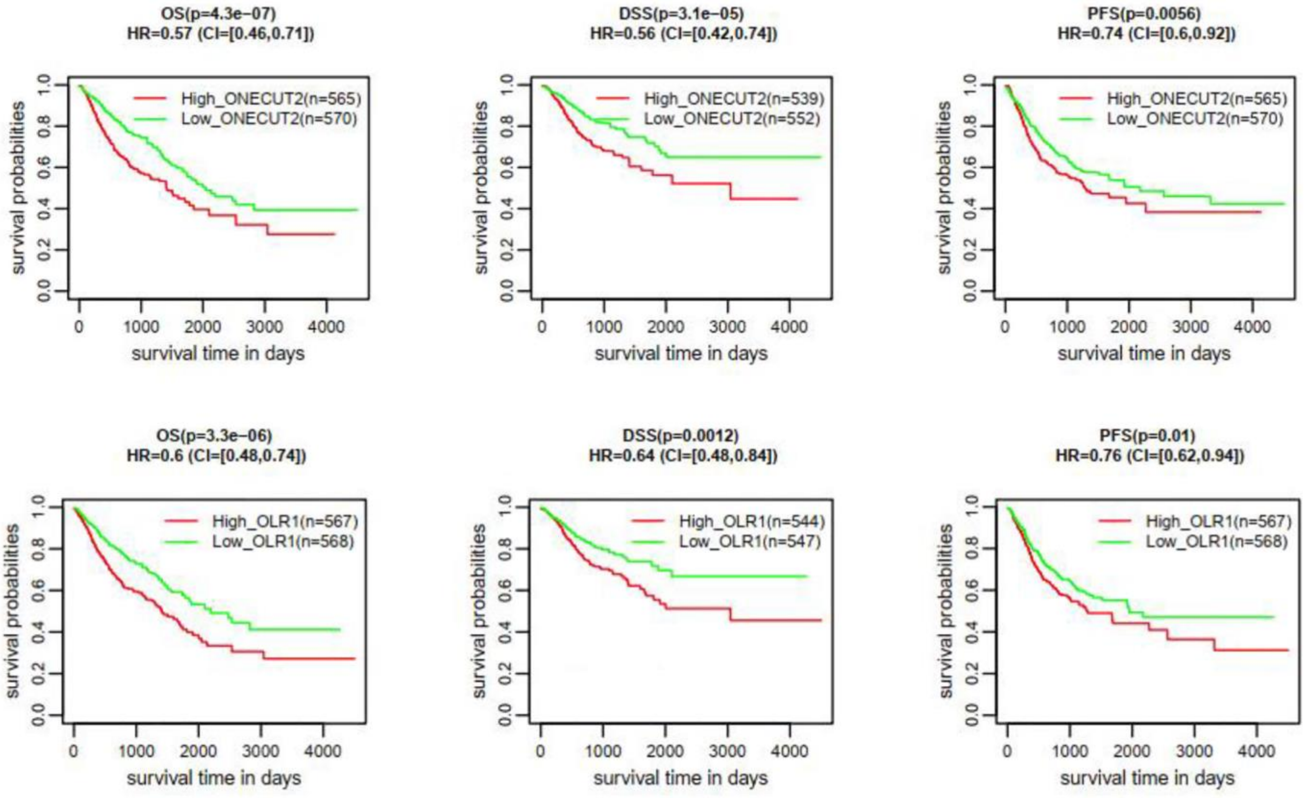

Supplementary Figure 6. The survival plot of patient groups with different OLR1 and ONECUT2 expression in TCGA GIAC cohort.

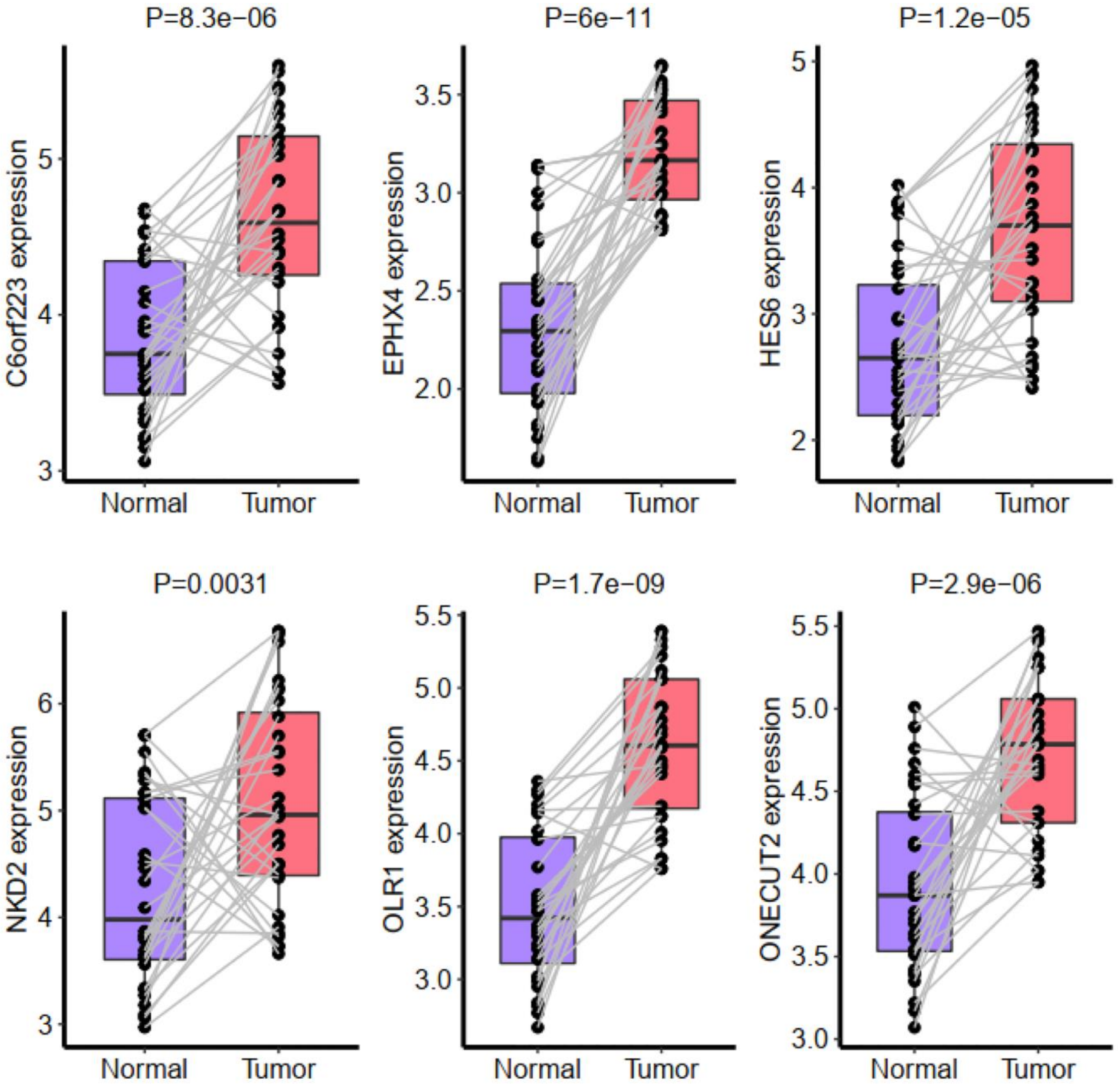

Supplementary Figure 7. The RNA expression of the six genes in 32 pairs of GAD tumor and normal samples from our center.

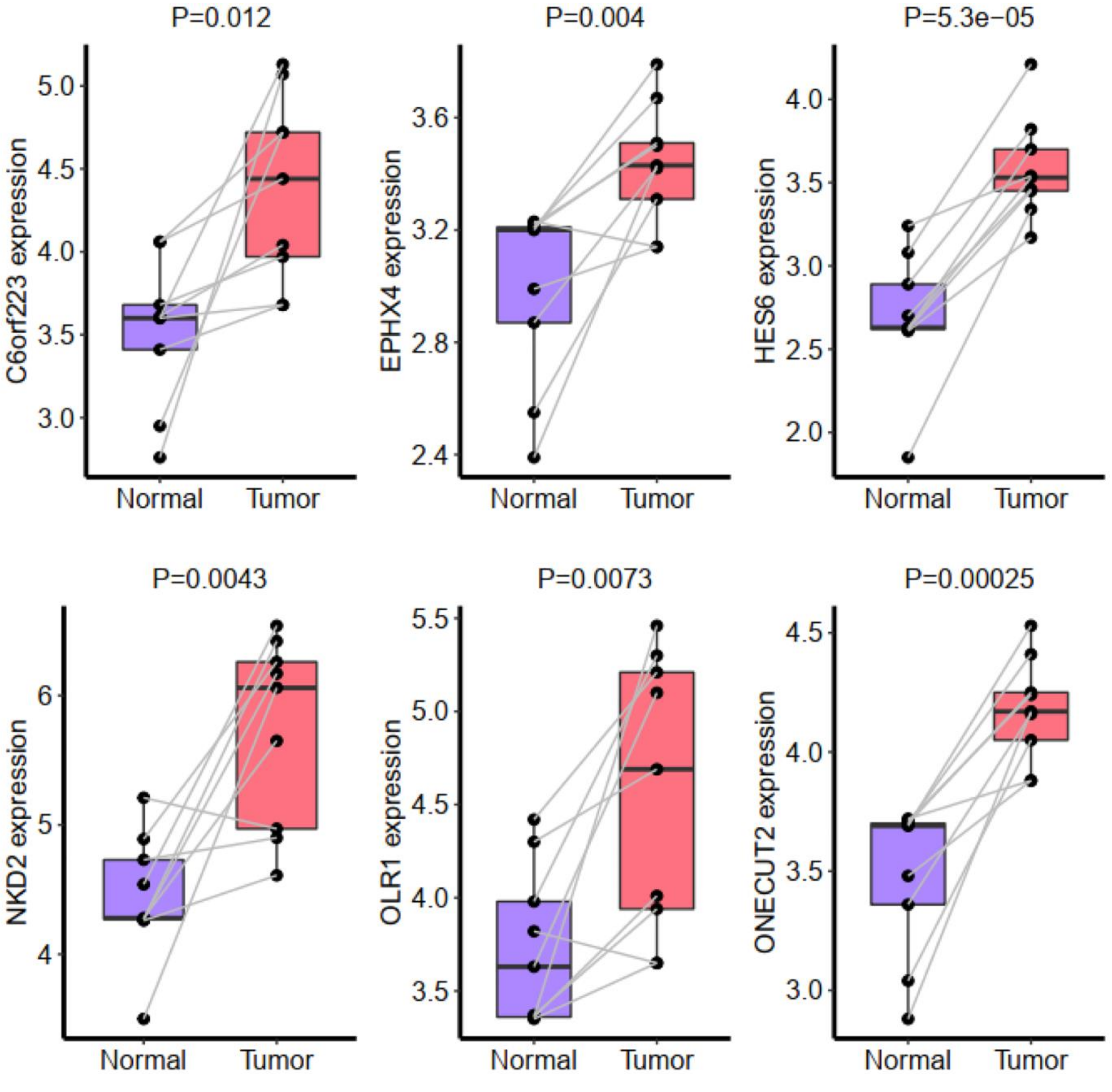

Supplementary Figure 8. The RNA expression of the six genes in 9 pairs of ESAD tumor and normal samples from our center.

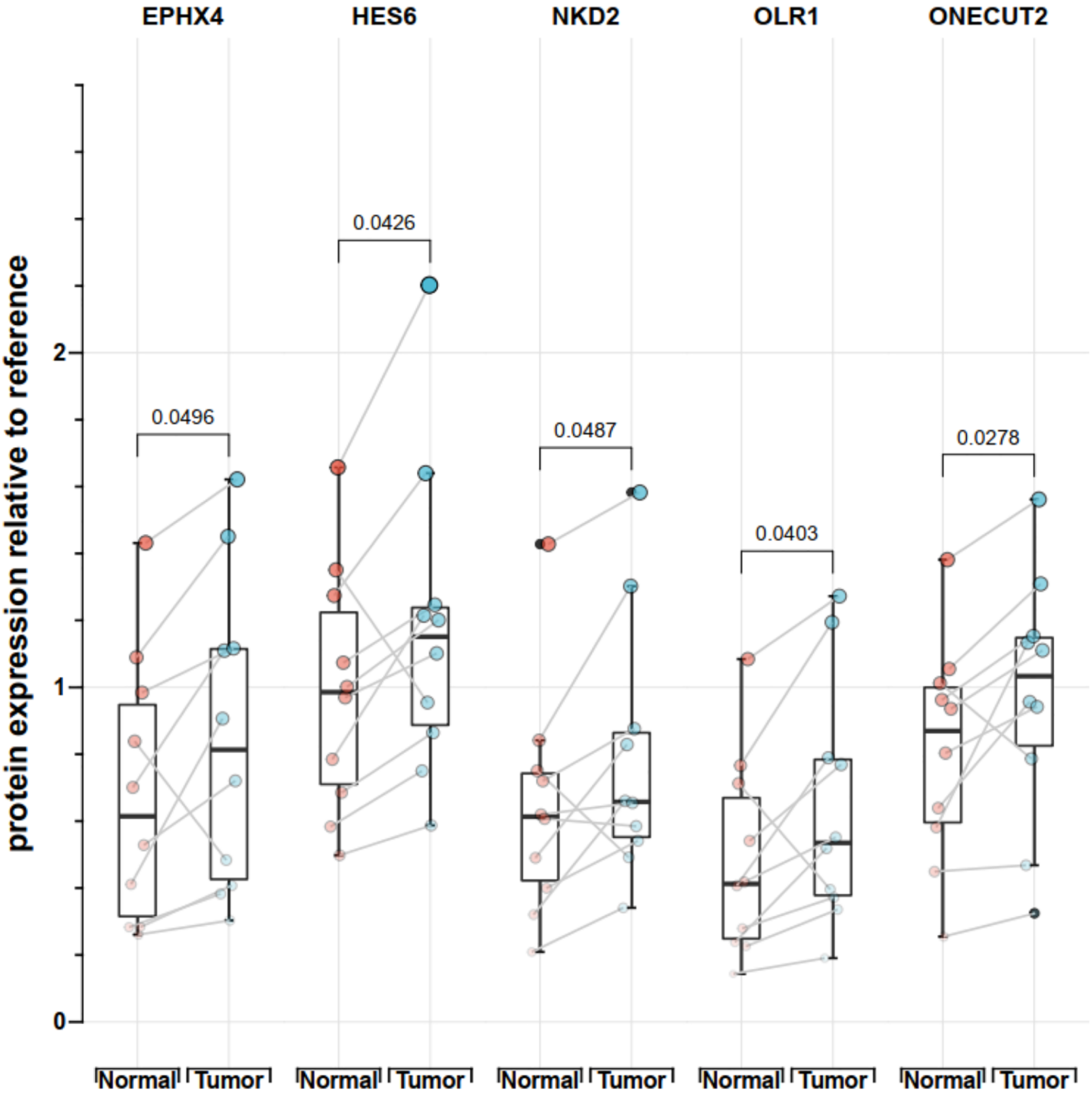

Supplementary Figure 9. The expression of the five proteins in 10 pairs of GAD tumor and normal samples from our center.

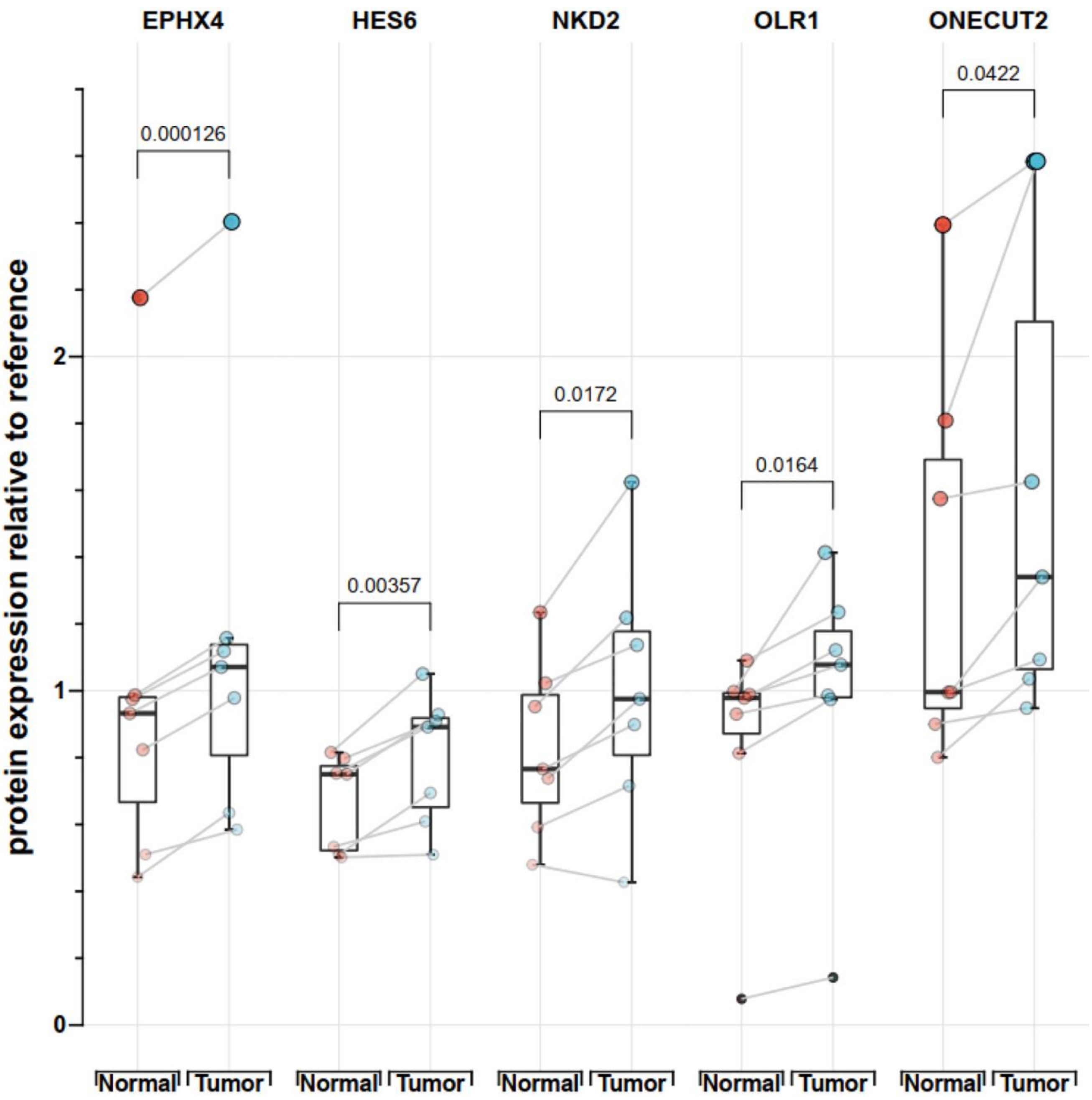

Supplementary Figure 10. The expression of the five proteins in 7 pairs of ESAD tumor and normal samples from our center.

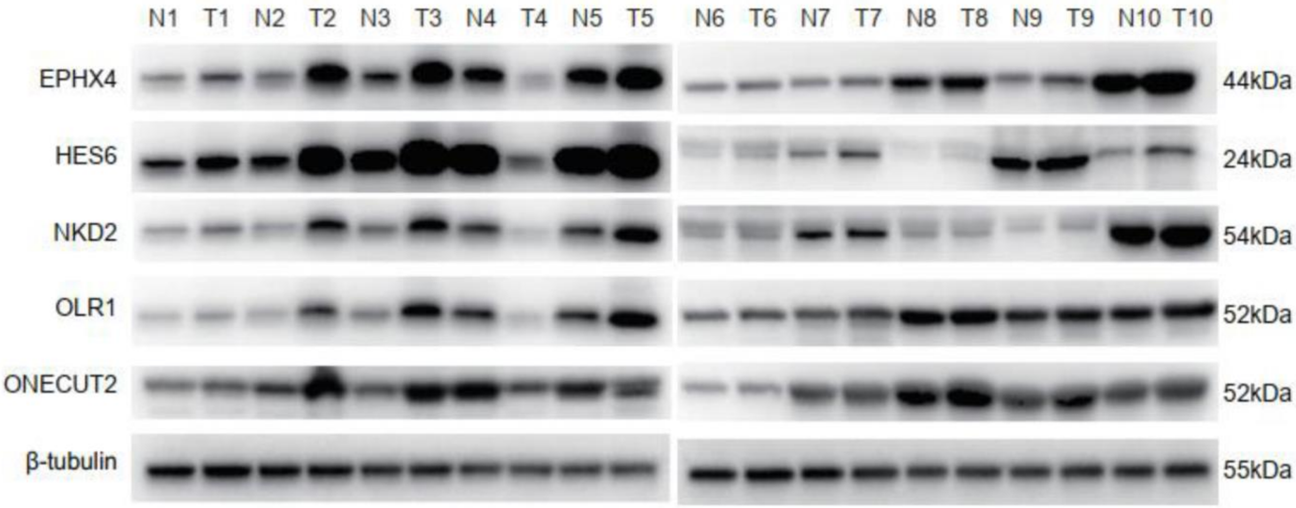

Supplementary Figure 11. The WB image of protein expression for GAD.

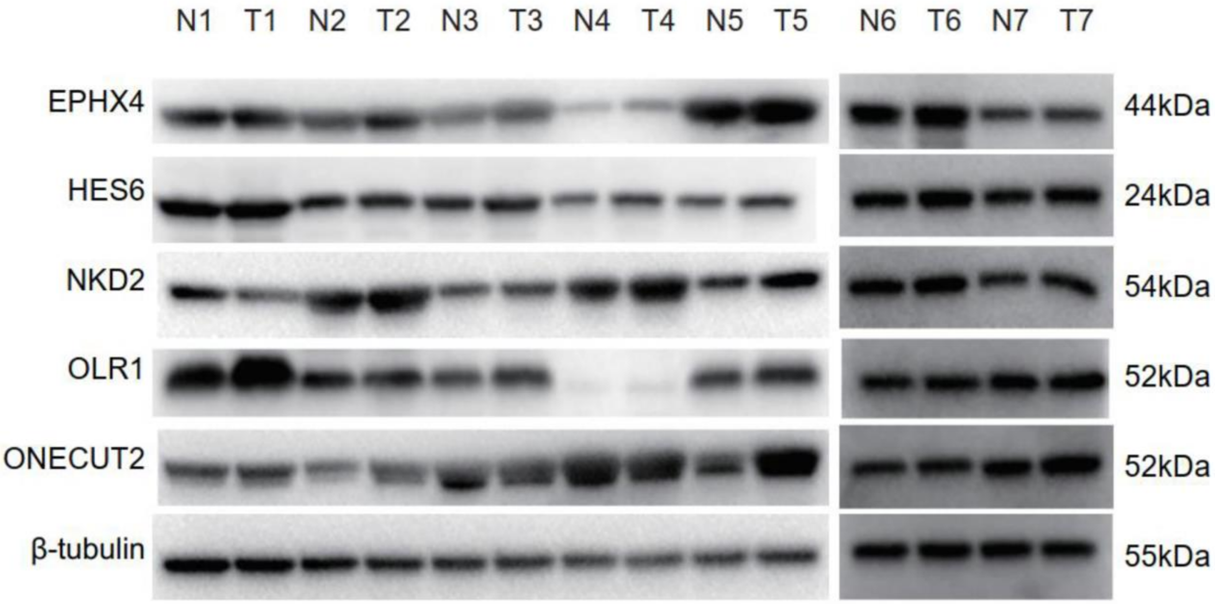

Supplementary Figure 12. The WB image of protein expression for ESAD.

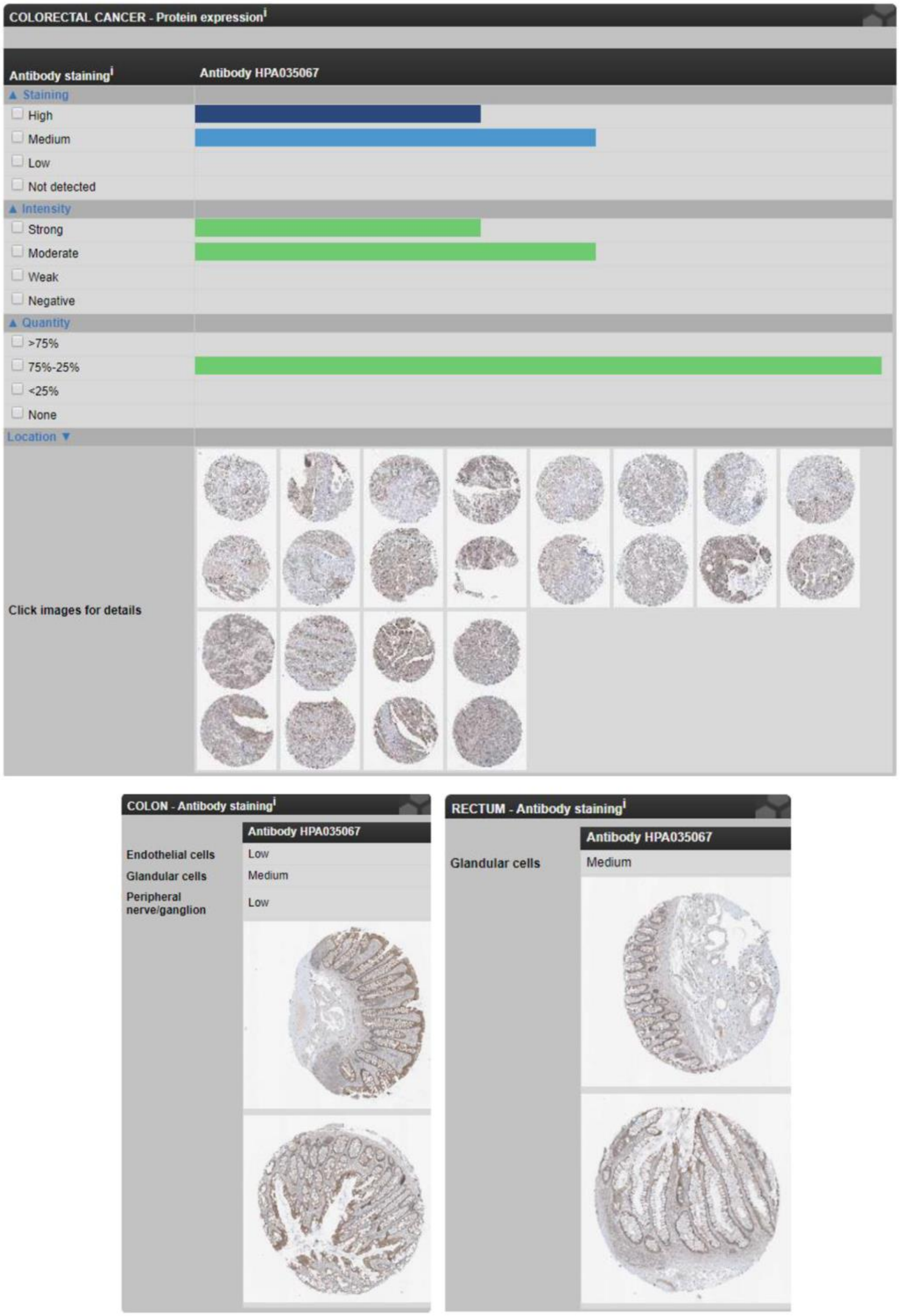

Supplementary Figure 13. EPHX4 staining of colorectal cancer and normal colon/rectum tissues in HPA database.

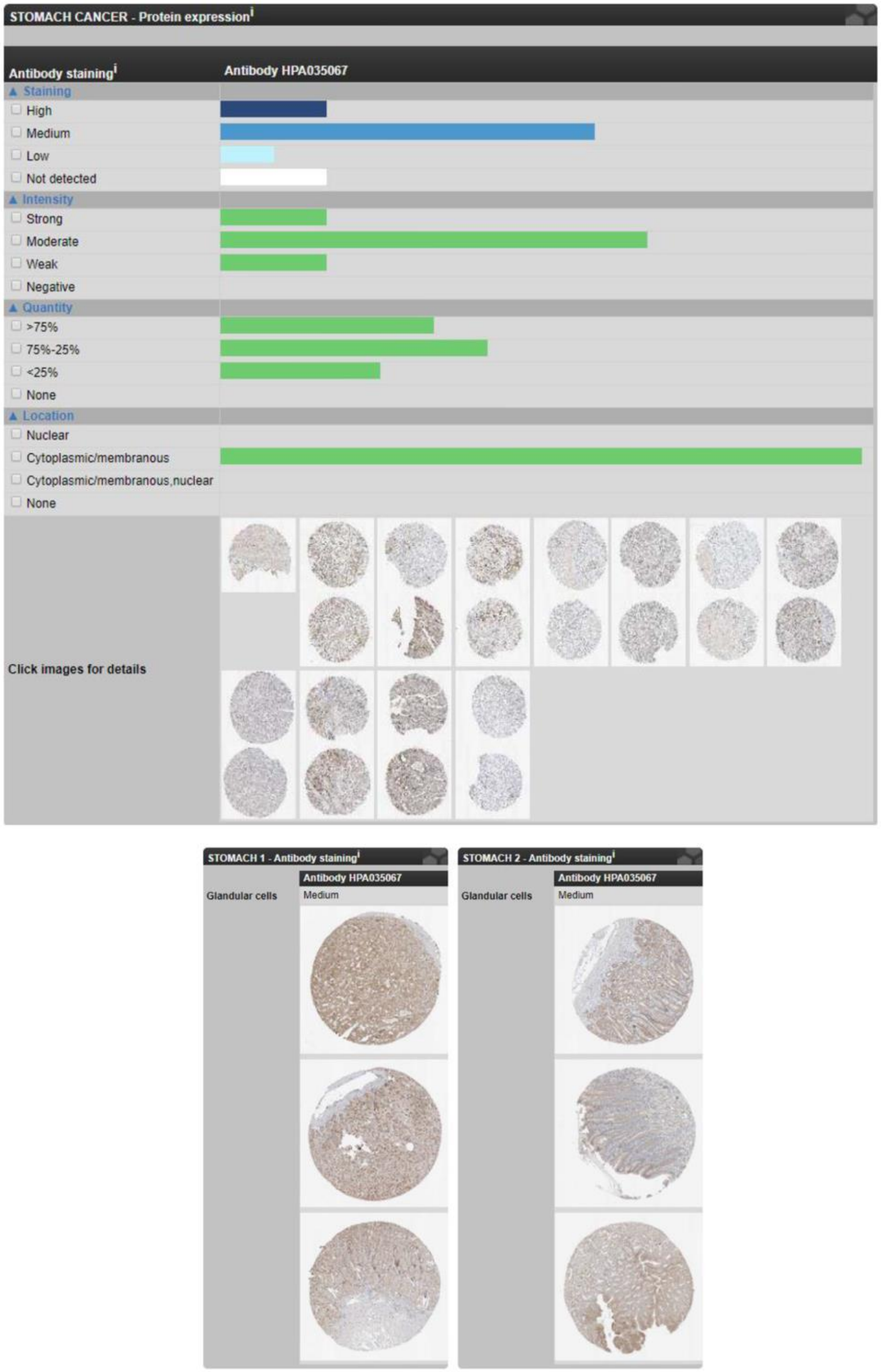

Supplementary Figure 14. EPHX4 staining of stomach cancer and normal stomach tissues in HPA database.

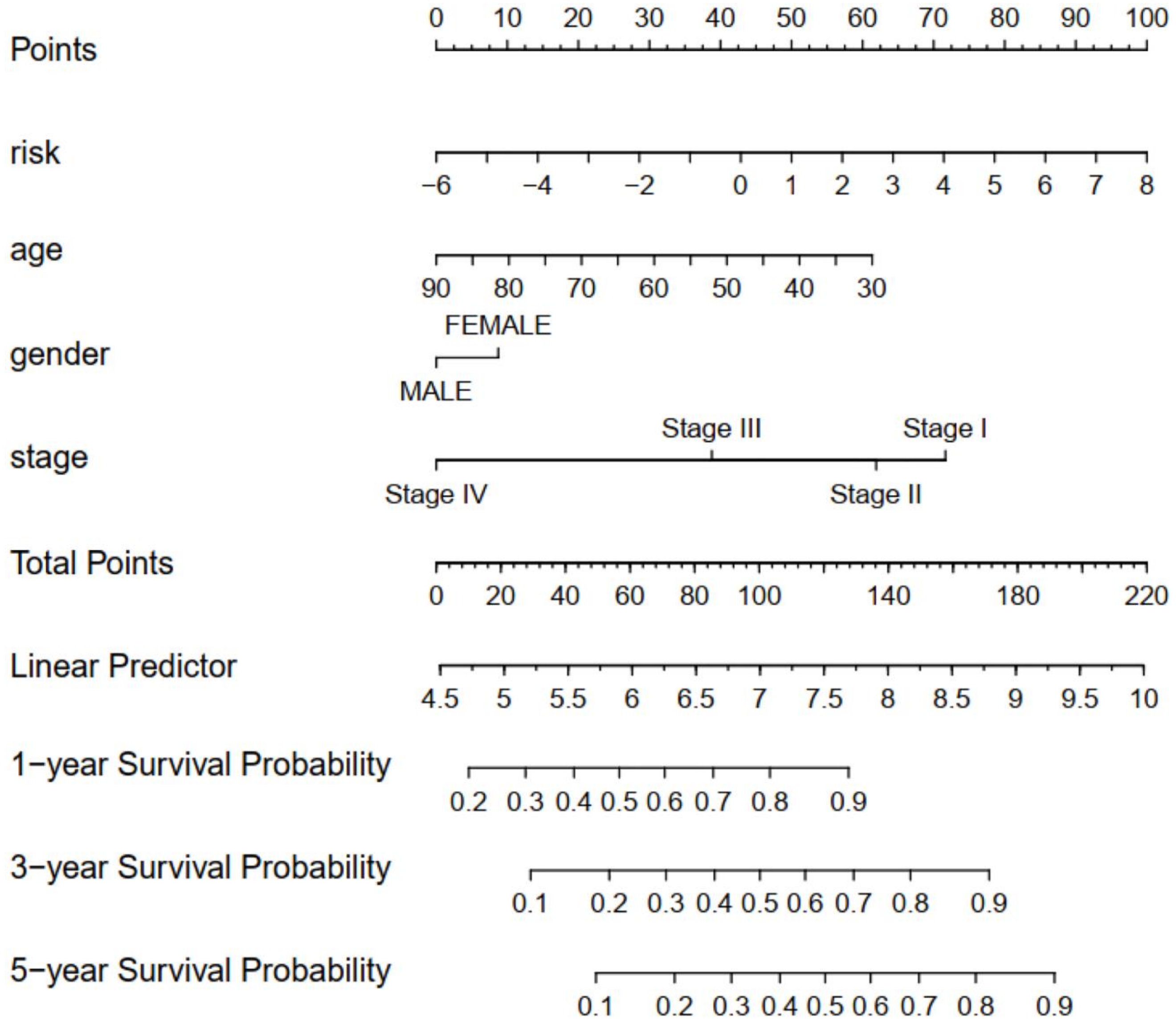

Supplementary Figure 15. The nomogram plot of TMEscore for predicting OS.

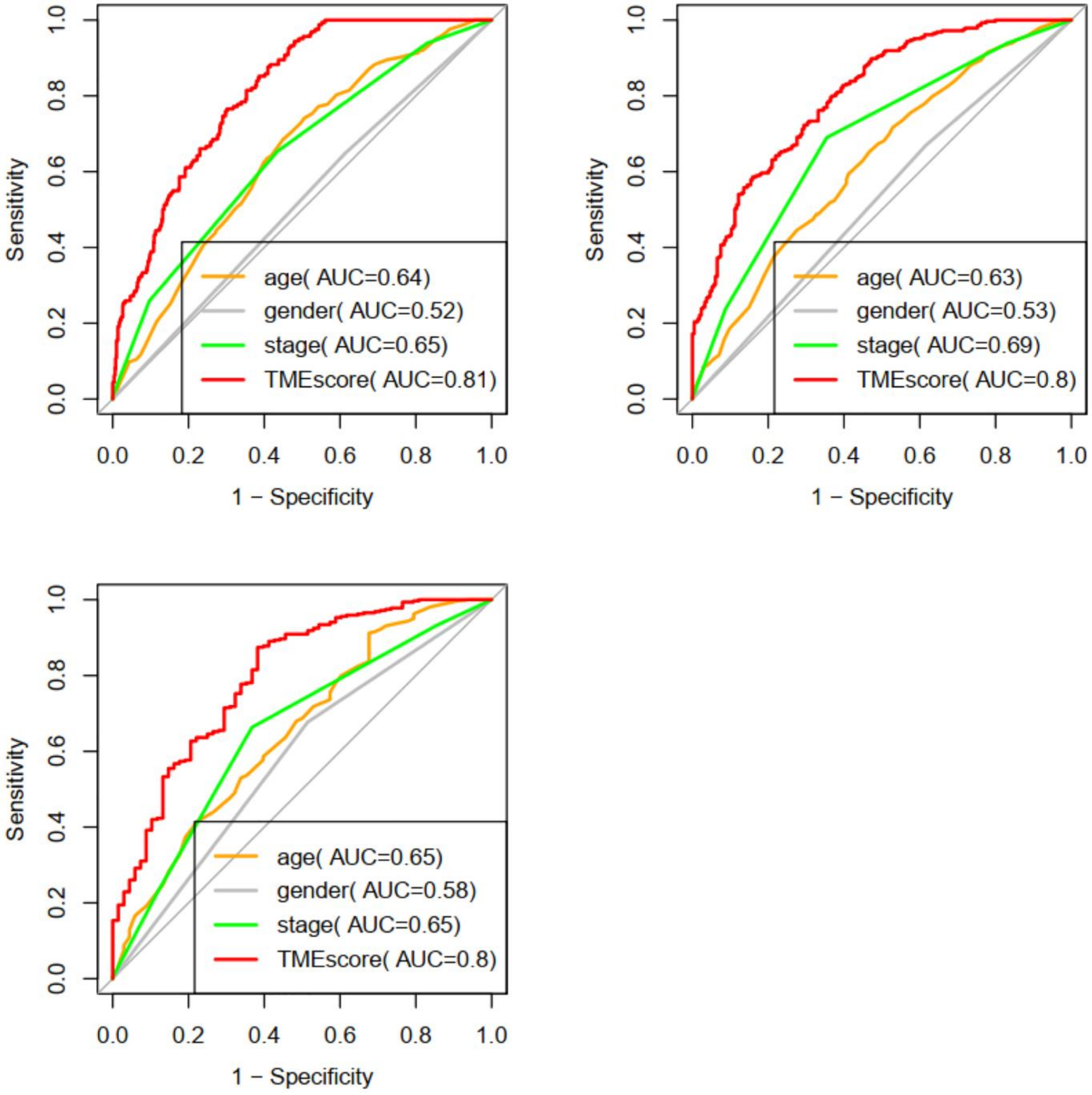

Supplementary Figure 16. The ROC curve plot of TMEscore for predicting OS compared to tumor stage.

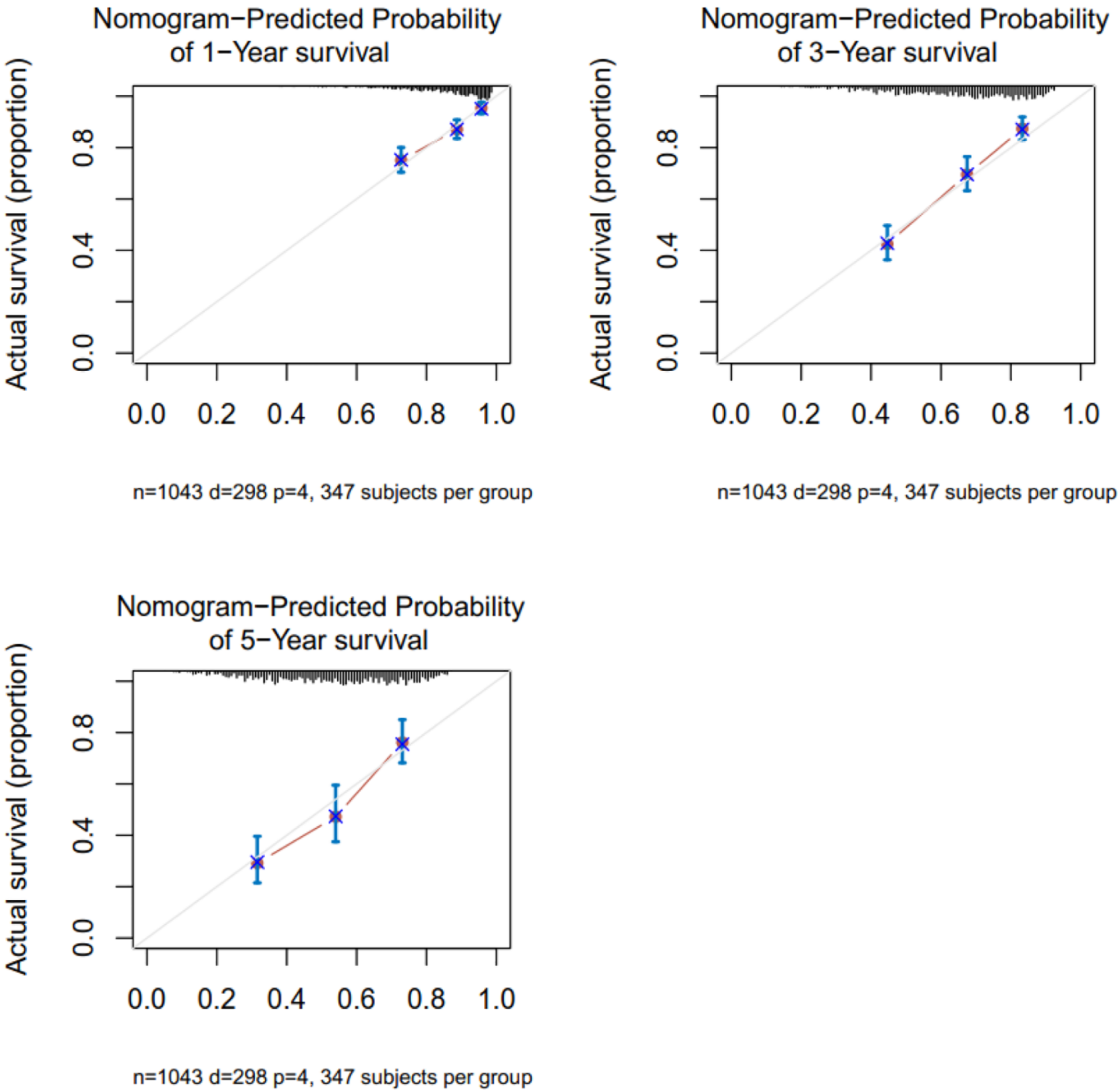

Supplementary Figure 17. The calibration plot of the model integrating TMEscore and tumor grade for predicting OS.

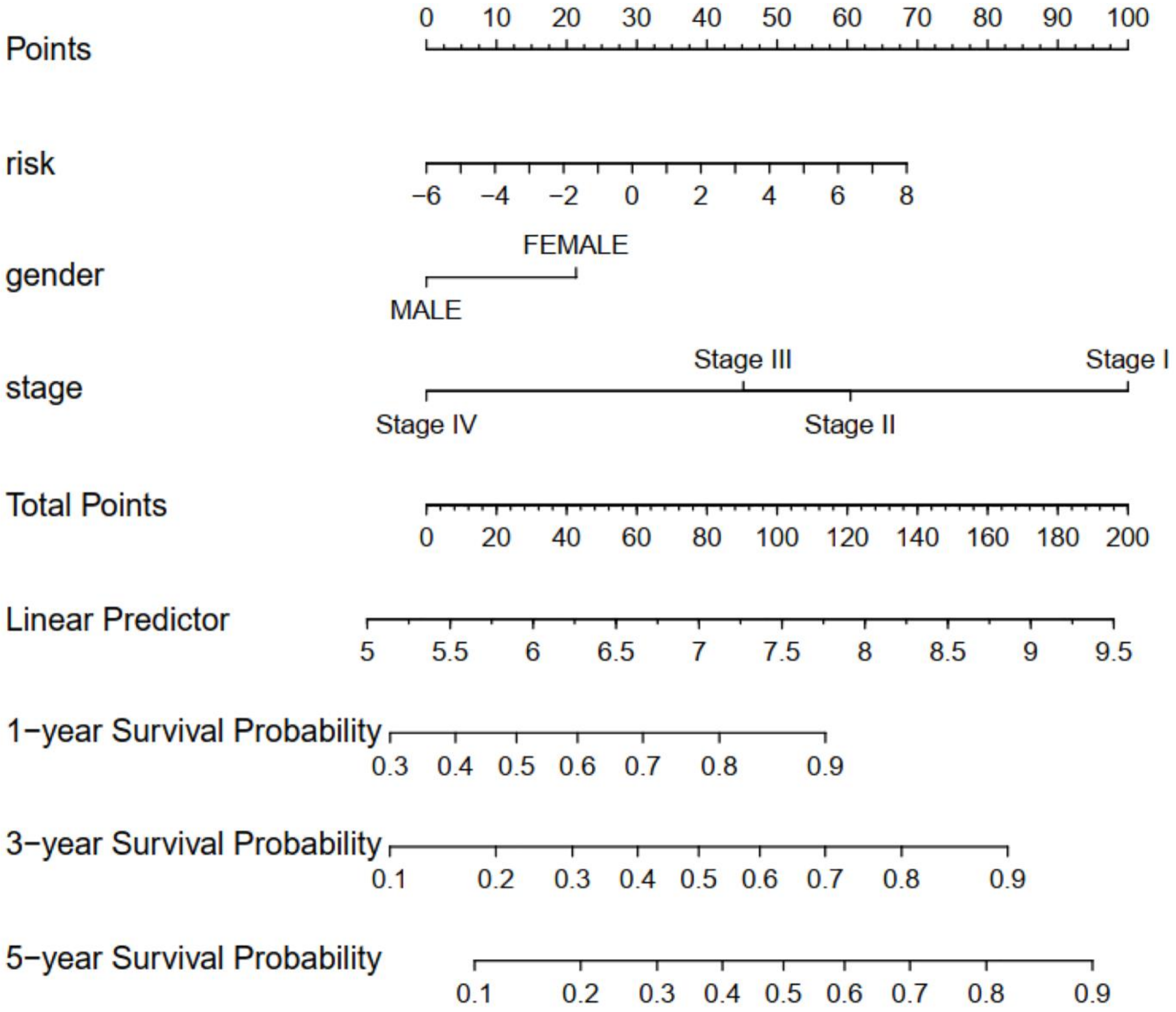

Supplementary Figure 18. The nomogram plot of TMEscore for predicting PFS.

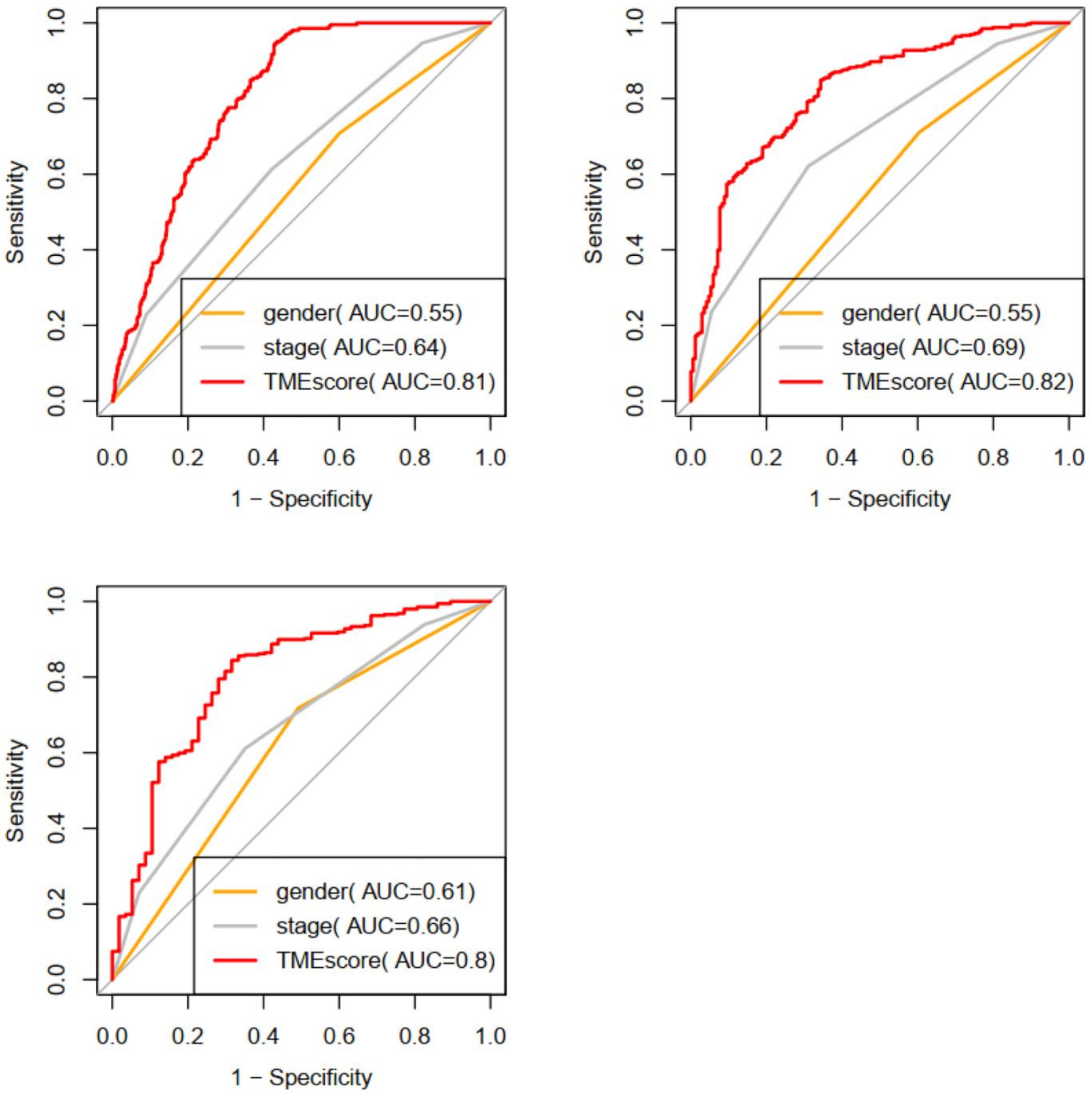

Supplementary Figure 19. The ROC curve plot of TMEscore for predicting PFS compared to tumor stage.

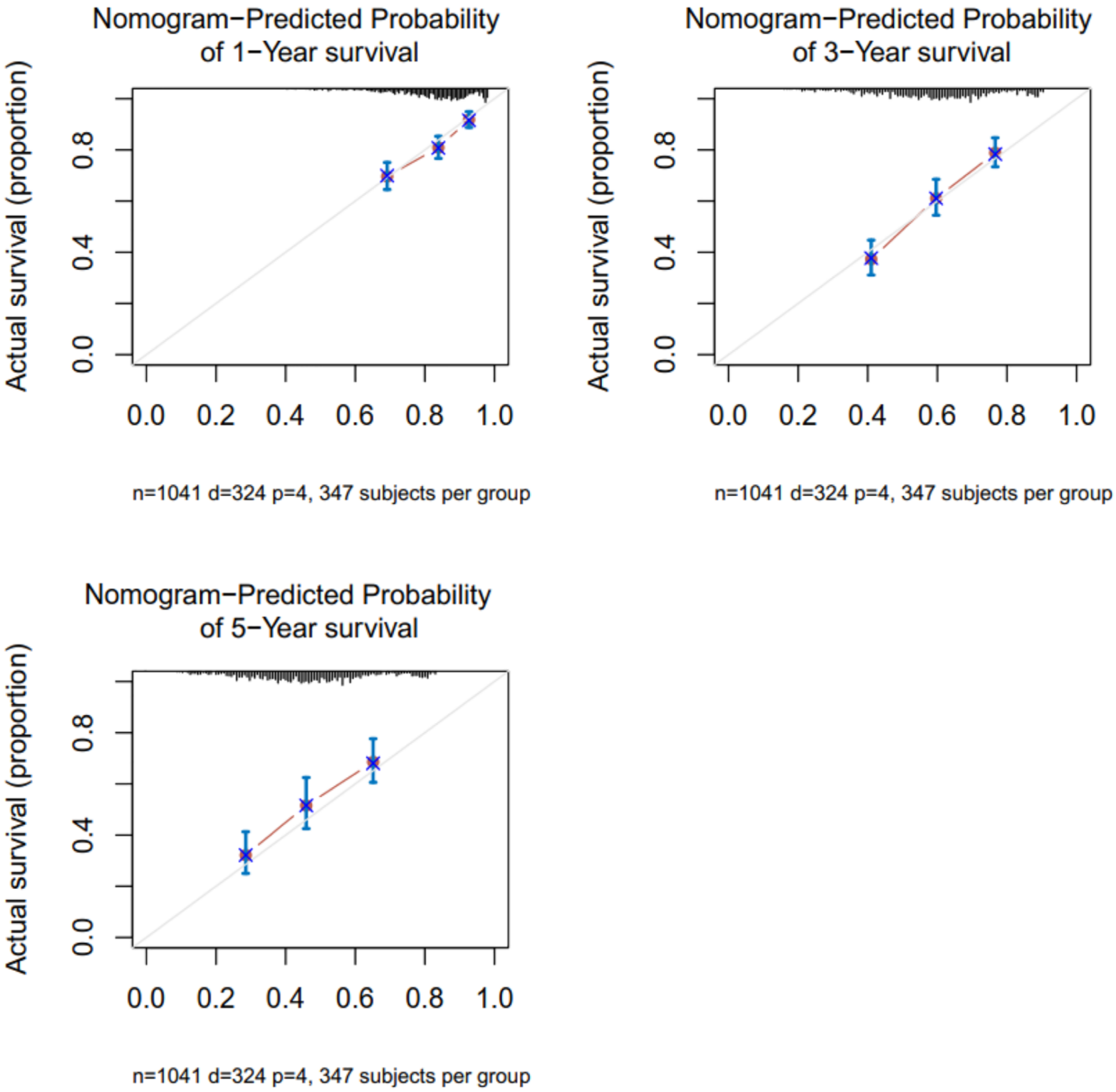

Supplementary Figure 20. The calibration plot of the model integrating TMEscore and tumor grade for predicting PFS.

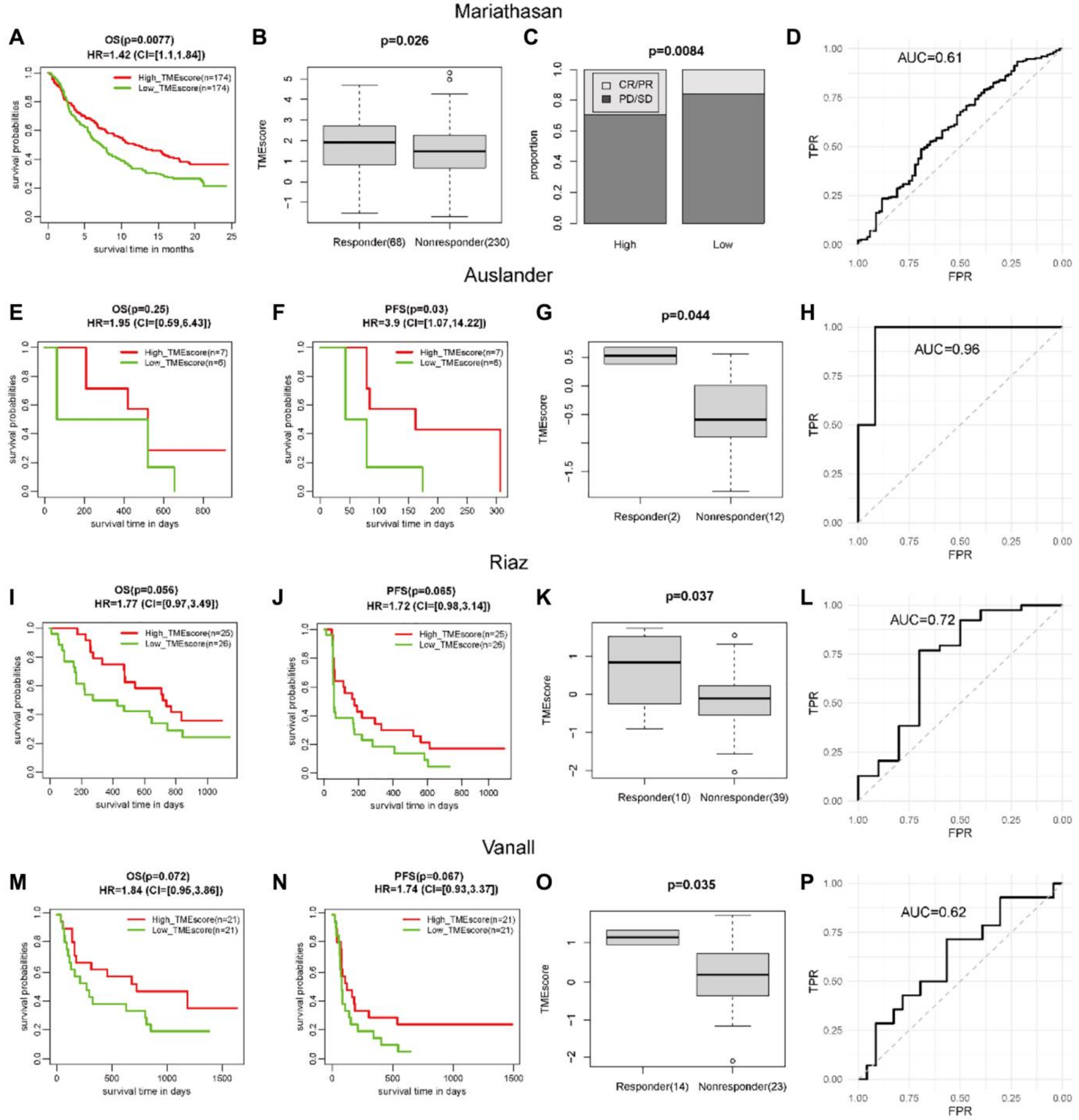

Supplementary Figure 21. TMEscore was a prognostic and immunotherapeutic biomarker in other four immunotherapeutic data sets. (**A**, **B**) The survival time and response rate of patients with high/low TMEscore in Mariathasan cohort. (**C**) The TMEscore of patients with different response status. (**D**) The ROC curve of TMEscore predicting immunotherapeutic response. (**E**, **F**) The survival time (OS and PFS) of patients with high/low TMEscore in Auslander cohort. (**G**) The TMEscore of patients with different response status (**H**) The ROC curve of TMEscore predicting immunotherapeutic response. (**I**, **J**) The survival time (OS and PFS) of patients with high/low TMEscore in Riaz cohort. (**K**) The TMEscore of patients with different response status (**L**) The ROC curve of TMEscore predicting immunotherapeutic response. (**M**, **N**) The survival time (OS and PFS) of patients with high/low TMEscore in Vanall cohort. (**O**) The TMEscore of patients with different response status. (**P**) The ROC curve of TMEscore predicting immunotherapeutic response.

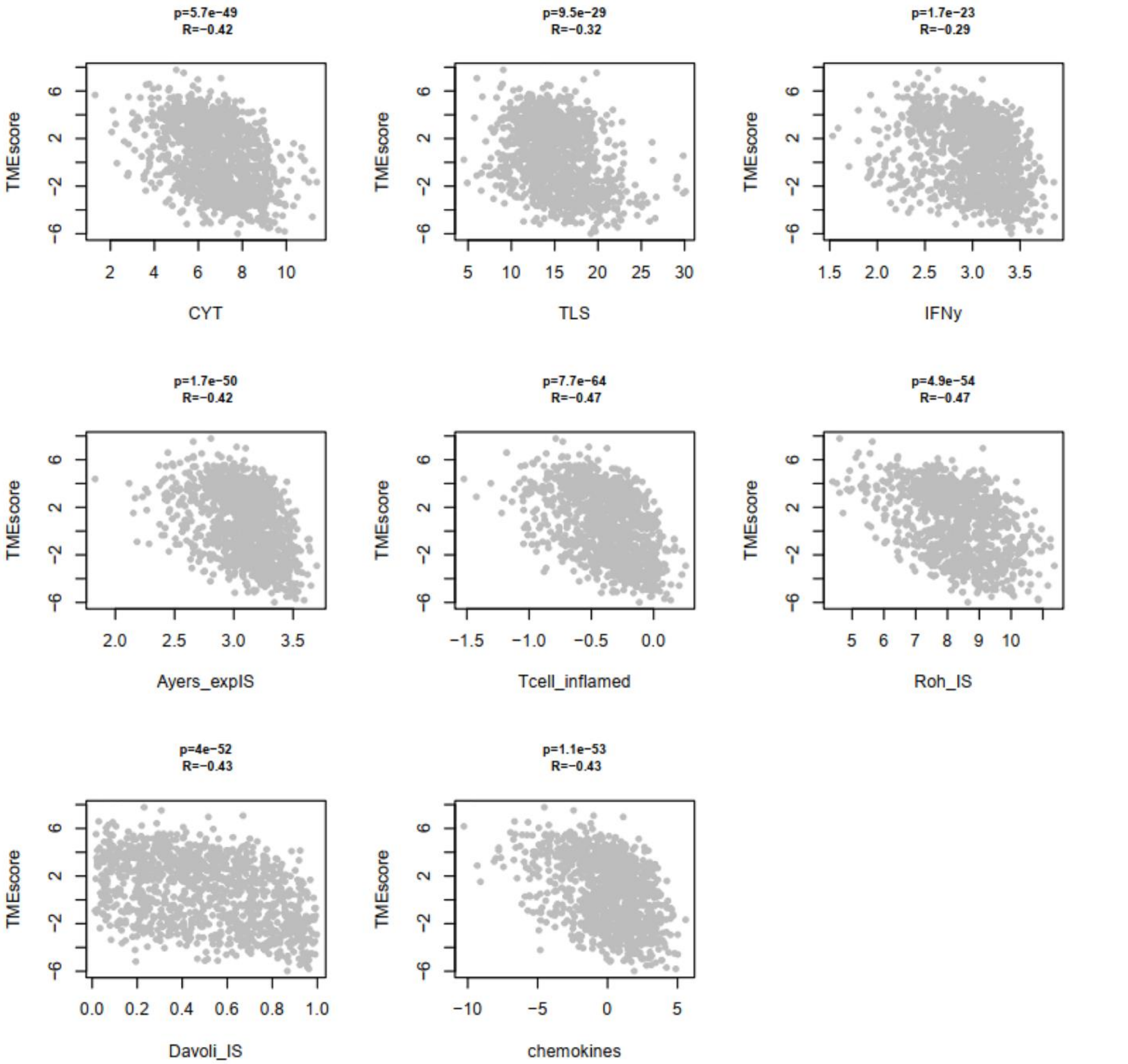

Supplementary Figure 22. The correlation plot of TMEscore with eight immune response scores estimated by "EaSIeR" in TCGA GIAC cohort.
