# Supplementary material for: The tumor microenvironment in gastrointestinal adenocarcinomas revealed a prognostic and immunotherapeutic biomarker
Source: Aging (Albany NY). 2022 Dec 30;14(24):10171–216. doi: 10.18632/aging.204463 (PMC9831739; doi:10.18632/aging.204463)
Supplement: Supplementary Tables 4, 9-11, 16, 19, 22-25 [file aging-14-204463-s003.pdf]

SUPPLEMENTARY TABLES

Supplementary Table 4. The primers used in this study.

Name	Direction	Sequence(5′–3′)
C6orf223	Forward	GCTGTGAGAGTTTTCCCCGA
	Reverse	CCTTTTGACCCCAAAGCACG
EPHX4	Forward	CCTGGTCTACTGCTACTGCG
	Reverse	TTTCAACAGCTGAGCAGGGT
HES6	Forward	CCGAGCTCCTGAACCATCTG
	Reverse	GCTGCAGGGTCCCTAAAAGT
NKD2	Forward	AAGTCCGGGAAAGCCTTCAG
	Reverse	AGATGTGTTCACCGCCATGT
OLR1	Forward	CATTATGGTGCTGGGCATGC
	Reverse	TGGGGCATCAAAGGAGAACC
ONECUT2	Forward	GGAATCCAAAACCGTGGAGTAA
	Reverse	CTCTTTGCGTTTGCACGCTG
GAPDH	Forward	CCGGGAAACTGTGGCGTGATGG
	Reverse	AGGTGGAGGAGTGGGTGTCGCTGTT

Supplementary Table 9. The statistic information about the infiltration level of 6 immune cell types in GIAC tumor and normal samples.

	B_cell	CD4_Tcell	CD8_Tcell	Neutrophil	Macrophage	Dendritic
Tumor (average)	0.07	0.14	0.15	0.11	0.04	0.48
Normal (average)	0.1	0.14	0.17	0.09	0.07	0.44
log2 (Fold change)	−0.46	\	−0.11	0.31	−0.73	0.15
p value	1.60E-06	1	0.033	4.70E-23	4.20E-06	4.80E-11

Supplementary Table 10. The infiltration correlation of 6 immune cell types in in GIAC tumor samples.

	B_cell	CD4_Tcell	CD8_Tcell	Neutrophil	Macrophage	Dendritic
B_cell	1	0.16	−0.18	−0.45	−0.08	−0.47
CD4_Tcell	0.16	1	−0.69	−0.3	−0.07	−0.07
CD8_Tcell	−0.18	−0.69	1	0	−0.08	−0.21
Neutrophil	−0.45	−0.3	0	1	−0.06	0.12
Macrophage	−0.08	−0.07	−0.08	−0.06	1	−0.55
Dendritic	−0.47	−0.07	−0.21	0.12	−0.55	1

Supplementary Table 11. The univariate Cox regression result of 6 immune cell types for OS, DSS and PFS in TCGA.

	Survival_index	HR	log10 (*p*-value)	Threshold	Percentage
B_cell	OS	1.2	2.7	non	3.64
CD4_Tcell	OS	0.81	3.8	Favorable	6.85
CD8_Tcell	OS	1.4	9	Risk	7.72

Neutrophil	OS	1.2	2.3	non	5.53
Macrophage	OS	0.91	1.3	non	2.18
Dendritic	OS	0.83	3.4	Favorable	24.09
B_cell	DSS	1	0.18	non	3.64
CD4_Tcell	DSS	0.66	7.8	Favorable	6.85
CD8_Tcell	DSS	1.6	9.1	Risk	7.72
Neutrophil	DSS	1.2	1.9	non	5.53
Macrophage	DSS	1	0.029	non	2.18
Dendritic	DSS	0.84	2.1	non	24.09
B_cell	PFS	1.1	1	non	3.64
CD4_Tcell	PFS	0.74	7.2	Favorable	6.85
CD8_Tcell	PFS	1.3	4.7	Risk	7.72
Neutrophil	PFS	1.3	4.9	Risk	5.53
Macrophage	PFS	0.86	2.6	non	2.18
Dendritic	PFS	1	0.029	non	24.09

Supplementary Table 16. The statistics of 6 immunotherapy targets' expression from three subgroups.

	TMEC1	TMEC2	TMEC3	*p*_value
BTLA	2.61	4.56	2.8	5.00E-55
CD274	4.31	5.83	4.1	1.00E-55
CTLA4	5.01	6.5	4.68	7.40E-67
HAVCR2	6.92	8.34	6.64	1.80E-66
LAG3	5.66	7.19	5.23	6.80E-63
PDCD1	4.46	5.92	4.24	6.40E-66

Supplementary Table 19. The *p*-value in functional enrichment of signature A and B on pan-immune genesets.

	gene_sigA	gene_sigB
PanImmune_GeneSet	1	1.30E-07
HER2_Immune_PCA_18006808	0.85	0.0061
CSR_Activated_15701700	1	1
GRANS_PCA_16704732	1	0.15
B_cell_PCA_16704732	1	0.51
CHANG_CORE_SERUM_RESPONSE_UP	1	1
T_cell_PCA_16704732	1	1
Troester_WoundSig_19887484	0.38	0.037
Module11_Prolif_score	1	1
CSF1_response	1	0.028
STAT1_score	1	1.60E-08
KEGG_HEMATOPOIETIC_CELL_LINEAGE	1	0.41
Module4_TcellBcell_score	1	0.39
TGFB_score_21050467	0.27	0.0011

Module5_TcellBcell_score	1	1.80E-07
Immune_cell_Cluster_21214954	1	7.20E-05
Immune_NSCLC_score	1	0.068
IFNG_score_21050467	1	1
LCK_19272155	1	1
Minterferon_Cluster_21214954	1	0.032
MCD3_CD8_21214954	1	1
LYMPHS_PCA_16704732	1	1
Angiogenesis	1	1
Tcm cells	1	1
CD8 T cells	1	1
Interferon_Cluster_21214954	1	0.19
NK cells	1	1
Macrophages	1	0.015
B cells	1	1
Tfh cells	1	1
Neutrophils	1	0.17
MDACC.FNA.1_20805453	1	1
iDC	1	0.17
Eosinophils	1	1
TAMsurr_TcClassII_ratio	1	0.00055
Mast cells	1	1
Th1 cells	1	0.15
Th2 cells	1	1
TcClassII_score	1	1
Bcell_21978456	1	1
Tcell_21978456	1	1
T helper cells	1	1
Module3_IFN_score	1	0.0078
ZHANG_INTERFERON_RESPONSE	1	1
IL12_score_21050467	1	1
IL2_score_21050467	1	1
IgG_19272155	1	1
ICR_SCORE	1	1.80E-08
T cells	1	1
CD8_PCA_16704732	1	1
LIexpression_score	1	1

Supplementary Table 22. The univariate Cox regression result of 6 genes for OS and PFS in TCGA.

Gene	Survival Index	HR	coef	*P* value
C6orf223	OS	2	0.68	2.30E-09
EPHX4	OS	1.9	0.62	4.50E-08

HES6	OS	2.2	0.79	6.00E-12
NKD2	OS	1.6	0.44	7.80E-05
ONECUT2	OS	0.57	-0.56	5.90E-07
OLR1	OS	0.6	-0.51	4.20E-06
C6orf223	PFS	1.6	0.48	7.80E-06
EPHX4	PFS	1.4	0.37	0.00062
HES6	PFS	2	0.68	4.50E-10
NKD2	PFS	1.2	0.15	0.016
ONECUT2	PFS	0.74	-0.29	0.0058
OLR1	PFS	0.76	-0.27	0.01

Supplementary Table 23. The high expression of the five genes in literatures.

Gene	PMID	Cancer Type
OLR1(LOX-1)	34921134	Colon Cancer
OLR1(LOX-1)	30483757	Gastric Cancer
OLR1(LOX-1)	28345638	Gastric Cancer
OLR1(LOX-1)	22641216	Colorectal Cancer
HES6	30015909	Colorectal Cancer
HES6	33681178	Colorectal Cancer
ONECUT2(OC2)	32801861	Gastric Cancer
ONECUT2(OC2)	34253241	Colorectal Cancer
ONECUT2(OC2)	35874630	Colorectal Cancer
ONECUT2(OC2)	32129880	Gastric Cancer
ONECUT2(OC2)	33015779	Gastric Cancer
ONECUT2(OC2)	34365839	Gastric Cancer
NKD2	33455110	Colon Cancer
NKD2	27246976	Gastric Cancer
NKD2	26396173	Gastric Cancer
NKD2	26985708	Colon Cancer
EPHX4	31404997	Rectal Cancer

Supplementary Table 24. The Cox regression result of TMEscore for OS in TCGA.

**Univariate Cox regression for OS**				
	**HR**	**lower .95**	**upper .95**	***P* value**
TMEscore	0.85	0.82	0.88	1.50E-16
age	1	1	1	0.0024
Gender = MALE (ref = FEMALE)	1.4	1.1	1.8	0.0024
Stage = II (ref=I)	1.6	1	2.7	0.04
Stage = III (ref=I)	3	1.9	4.8	2.80E-06
Stage = IV (ref=I)	5.5	3.4	9	9.20E-12
**Multi-variate Cox regression for OS**				
	**HR**	**lower .95**	**upper .95**	***P* value**

TMEscore	0.84	0.8	0.88	4.60E-14
age	1	1	1	6.90E-09
Gender = MALE (ref = FEMALE)	1.1	0.84	1.4	0.51
Stage = II (ref = I)	2	1.1	3.8	0.023
Stage = III (ref = I)	3.7	2	6.8	1.70E-05
Stage = IV (ref = I)	10	5.5	19	1.80E-13

Supplementary Table 25. The Cox regression result of TMEscore for PFS in TCGA.

**Univariate Cox regression for PFS**				
	**HR**	**lower .95**	**upper .95**	***P* value**
TMEscore	0.9	0.87	0.93	2.80E-08
age	0.99	0.98	1	0.14
Gender = MALE (ref = FEMALE)	1.7	1.4	2.2	5.50E-06
Stage = II (ref = I)	2.4	1.5	3.8	0.00039
Stage = III (ref = I)	3.3	2.1	5.3	5.70E-07
Stage = IV (ref = I)	7.6	4.6	12	9.50E-16
**Multi-variate Cox regression for PFS**				
	**HR**	**lower .95**	**upper .95**	***P* value**
TMEscore	0.91	0.87	0.95	3.70E-05
Gender = MALE (ref = FEMALE)	1.4	1.1	1.8	0.0086
Stage = II (ref = I)	2.3	1.3	4	0.0026
Stage = III (ref = I)	3.2	1.8	5.5	3.70E-05
Stage = IV (ref = I)	8.3	4.8	15	1.20E-13
